# Lipidomics and mass spectrometry imaging unveil alterations in mice hippocampus lipid composition exposed to hypoxia

**DOI:** 10.1016/j.jlr.2024.100575

**Published:** 2024-06-10

**Authors:** Jiayue Gao, Zhiying Guo, Ming Zhao, Xiang Cheng, Xiufang Jiang, Yikun Liu, Wenpeng Zhang, Xiangpei Yue, Xuechao Fei, Yaqun Jiang, Lu Chen, Shaojie Zhang, Tong Zhao, Lingling Zhu

**Affiliations:** 1 Department of Brain Plasticity, Beijing Institute of Basic Medical Sciences, Beijing, China; 2 Hepato-pancreato-biliary Center, Beijing Tsinghua Changgung Hospital, Tsinghua University, Beijing, China; 3 State Key Laboratory of Precision Measurement Technology and Instruments, Department of Precision Instrument, Tsinghua University, Beijing, China; 4 Department of Gastroenterology, The Second Medical Center & National Clinical Research Center for Geriatric Diseases, Chinese PLA General Hospital, Beijing, China; 5 Co-Innovation Center of Neuroregeneration, Nantong University, Nantong, China

**Keywords:** LC-MS, lipidomics, lipidome, MSI, hypobaric hypoxia

## Abstract

Lipids are components of cytomembranes that are involved in various biochemical processes. High-altitude hypoxic environments not only affect the body’s energy metabolism, but these environments can also cause abnormal lipid metabolism involved in the hypoxia-induced cognitive impairment. Thus, comprehensive lipidomic profiling of the brain tissue is an essential step toward understanding the mechanism of cognitive impairment induced by hypoxic exposure. In the present study, mice showed reduced new-object recognition and spatial memory when exposed to hypobaric hypoxia for 1 day. Histomorphological staining revealed significant morphological and structural damage to the hippocampal tissue, along with prolonged exposure to hypobaric hypoxia. Dynamic lipidomics of the mouse hippocampus showed a significant shift in both the type and distribution of phospholipids, as verified by spatial lipid mapping. Collectively, a diverse and dynamic lipid composition in mice hippocampus was uncovered, which deepens our understanding of biochemical changes during sustained hypoxic exposure and could provide new insights into the cognitive decline induced by high-altitude hypoxia exposure.

An increasing number of people visit high-altitude regions for various reasons, such as sports training, tourism, scientific research, work, and military operations (1). However, these high-altitude environments also pose challenges to human health. The brain is extremely sensitive to hypoxia and is the first organ to be compromised when compensatory mechanisms of the body are inadequate (2, 3). Cognitive functions, including visual, working memory, and executive functions, significantly decrease with increasing altitude (4, 5). It has also been reported that prolonged exposure to high-altitude hypoxia impairs attention, information processing rate, spatial cognitive ability, and executive functioning to varying degrees (2, 6, 7). In rodent models, acute hypoxia exposure or chronic hypobaric hypoxia exposure has been shown to induce cognitive impairment in terms of more transient associative, spatial memory impairments, and working memory alterations (8, 9, 10, 11). Overall, the severity of the cognitive deficit correlates with the duration and degree of hypoxia.

Exposure to hypoxia causes significant and comprehensive biological processes, including energy homeostasis and metabolites production. Lipid metabolism, as a major class of biological molecules, is also affected by hypoxic exposure. An epidemiological study of Tibetan populations living at 3660 m above sea level in Lhasa, Tibet, pointed to the presence of abnormal lipid levels, including triglycerides, total cholesterol, low-density lipoprotein cholesterol, and high-density lipoprotein (12). It has been reported that fatty acids (tetradecanoic acid, hexadecenoic acid, octadecenoic acid, linoleate, and so on) in erythrocyte extracts are upregulated after 16 days at 5260 m (13). Plasma metabolomic analysis also revealed that fatty acids (arachidonic acid, palmitic acid, linoleic acid, and oleic acid) and fatty acyl carnitines increased, and lyso-phosphatidylcholines (LPC) (LPC (18:0), LPC (18:2), and LPC (20:2)) decreased after arriving a high altitude (5300m) for four days (14). In addition, our previous study found that some phospholipids in plasma were included in the differential metabolites when ascending to a high-altitude plateau for three days (15). Similar changes were observed in the animal models. In the plasma of rats exposed to chronic hypobaric hypoxia for 30 days (4600m), total cholesterol, LDL cholesterol, VLDL cholesterol, and triglycerides were found to have increased, whereas HDL cholesterol decreased (16). It has been reported that the levels of phosphatidylinositol (PI) and phosphatidylserine (PS) decrease in the rat brains subjected to hypoxia decreased (17). Triacylglyceride (TAG) concentration was significantly increased in the hypoxic liver but remained unchanged in the brains of 7-day-old rats exposed to hypoxia from birth (18). Lipid supplementation has also been reported to improve cognitive function. Dietary PS supplements have been reported to improve cognitive function in experimental animals (19, 20), and a similar result was obtained with krill PS in rats (21, 22, 23). Similarly, cognitive functions are effectively supported in humans by oral PS at 200–800 mg/day (24, 25). These reports suggested that the disruption of lipid metabolism in the brain induced by hypobaric hypoxia may be a key factor in cognitive impairment. Therefore, it is important to obtain retrospective and prospective comprehensive profiles of the lipidome in the mouse brain to understand which and how lipid species could be altered by the hypoxia exposure and to further provide new insights into cognitive dysfunction induced by lipid metabolic disorders.

With the aim of providing a comprehensive lipidomic characterization of the mouse hippocampus exposed to a hypoxic environment and to unveil the lipid dynamics during hypoxia exposure, nontargeted lipidomics and MS imaging (MSI) on mouse hippocampal regions exposed to different hypoxia times were performed. To reveal the in situ localization of various lipids in the brain, mouse brain sections were examined using AP-MALDI-MSI. Our findings provide compositional information on lipids within the hippocampal tissue of mice and also spatial information on lipids in the brains of mice exposed to hypobaric hypoxia, in which the disruption of lipid metabolism might contribute to cognitive impairment.

## Materials and methods

### Animals model

The mice were purchased from the Laboratory Animal Center of the Vital River Experimental Animal Company (Beijing, China). In all of the experiments undertaken, the ethics guidelines for investigations involving conscious animals were followed, and the experiments were approved by the Animal Care and Use Committee of the Institute of Basic Medical Sciences. Eight-week-old male C57BL/6 mice weighing 18 ± 2 g (8 weeks old) were kept in a 12-h light/dark cycle with free access to standard rodent chow and water. Mice were maintained under specific pathogen-free conditions.

Hypoxia exposure studies in mice have mainly included short-, mid-, and long-term experiments. The acute phase was defined as one or more exposures within 24 h. By considering the fact that 24 h may not be sufficient to evaluate the time response of prolonged exposure to hypoxia, we set up a one-week period to investigate the effect of hypoxia exposure on the mouse brain. The mice were divided into four groups (n = 11 per group): a control group (Con), a group exposed to hypobaric hypoxia for 1 day (Hy1d), a group exposed to hypobaric hypoxia for 3 days (Hy3d), and a group exposed to hypobaric hypoxia for 7 days (Hy7d). Except for the Con group, all hypobaric hypoxia groups were placed in a hypobaric hypoxia chamber that simulated different altitudes. The mice were subjected to hypobaric hypoxia condition at an altitude of 7000 m (equivalent to 9.75% O_2_ at sea level) at a speed of 30 m/s for different durations. The mice were then descended to sea level at a speed of 30 m/s. The control group was housed under normobaric normoxia, whereas the other conditions studied were similar to those of the hypobaric hypoxia groups. Finally, the mice were euthanized after exiting the chamber, and the brain tissues were harvested immediately.

### Novel object recognition test

The novel object recognition test was performed in a clear box (50 cm × 50 cm × 20 cm). The mice acclimatized to the box for three days. The test comprised two phases. Phase 1 was a 5-min familiarization period, in which the animals were presented with two identical objects. Phase 2 was a 5-min test in which one familiar object was replaced by a novel object. The ANY-maze software (Panlab, Harvard Apparatus, https://www.any-maze.com/) was used to record and analyze the data. The time spent sniffing the novel and known objects was recorded.

### Morris water maze test

The water maze test consisted of two phases: (1) the training phase, 5 days with a hidden platform (four trials/day), and 2) the testing phase, during which the platform was removed from the maze, which lasted for 1 min, and was performed to assess the retention of previously acquired information. Mice were tracked using a video camera during training and testing. The collected data were analyzed using ANY-maze software.

### Morphological analysis of brain samples

Mouse brain samples of mice were fixed in 4% paraformaldehyde and then embedded in paraffin. Subsequently, 5 μm thickness sections were stained with Nissl staining and H&E staining if order to evaluate morphological variation with hypoxia treatment. All pathological changes in neurons of the hippocampal cornu ammonis 1 (CA1), cornu ammonis 3 (CA3), and dentate gyrus (DG) regions were observed under a microscope (Thermo Fisher Scientific).

### Quantitative real-time PCR

RNA from the hippocampus was extracted using TRIzol reagent (Invitrogen). Reverse transcription was performed using the Reverse Transcription Kit (Vazyme Biotech Co.). cDNA was amplified using SYBR qPCR Master Mix (Vazyme Biotech Co., China) in a thermal cycler (Bio-Rad). The primer sequences are listed in Table S1.

### Lipid extraction

Hippocampal tissues from four mice were used for extraction of each sample. A total of 100 mg of tissue from each brain hippocampus were thoroughly homogenized in 750 μl of −20°C cold methanol. The homogenate was vortexed for 10 s. Methyl tert-butyl ether (2.5 ml) was added and the mixture was vortexed for 10 s and then incubated for 1 h at room temperature in a shaker. Phase separation was induced by adding 0.625 ml of MS-grade water. After 10 min of incubation at room temperature, the samples were centrifuged at 1,000 *g* for 10 min. The upper (organic) phase was collected, and then the lower phase was reextracted with 1 ml of a solvent mixture methyl tert-butyl ether /methanol/water (10:3:2.5, v/v/v)), and the upper phase was collected. The combined organic phases were dried and dissolved in 100 μl of isopropanol for storage.

### UHPLC-MS/MS analysis

UHPLC-MS/MS analyses were performed using a Vanquish UHPLC system (Thermo Fisher Scientific, Germany) coupled with an Orbitrap Q Exactive™ HF mass spectrometer (Thermo Fisher Scientific, Germany) at Novogene Co., Ltd. (Beijing, China). Samples were injected onto a Thermo Accucore C30 column (150 × 2.1 mm, 2.6 μm) using a 20-min linear gradient at a flow rate of 0.35 ml/min. The column temperature was set at 40°C. Mobile phase buffer A consisted of acetonitrile: water, 6:4 (v/v) with 10 mM ammonium acetate and 0.1% formic acid, whereas buffer B consisted of acetonitrile: isopropanol, 1:9 (v/v) with 10 mM ammonium acetate and 0.1% formic acid. The solvent gradient was set as follows: 30% B, initial; 30% B, 2 min; 43% B, 5 min; 55% B, 5.1 min; 70% B, 11 min; 99% B, 16 min; and 30% B, 18.1 min. Q Exactive™ HF mass spectrometer was operated in the positive [negative] polarity mode with sheath gas: 20 arbitrary units, sweep gas: 1 arbitrary units, auxiliary gas rate: 5, spray voltage: 3 kV, capillary temperature: 350°C, Aux gas heater temperature: 400°C, S-Lens radio frequency level: 50, resolving power（full-scan）: 120,000, scan range: 114–1700 *m/z*, automatic gain control target: 1e6, resolving power（MS^2^）: 30,000（Top20）, normalized collision energy: 25; 30, injection time: 100 ms, isolation window: 1.0 m/z, automatic gain control target （MS^2^）: 1e5, dynamic exclusion: 15s.

### Sample preparation and section

The whole brain of a mouse was removed and then immediately stored at −80°C for further analysis. The mouse brain samples were cut into 10 μm thickness sections with a cryotome at −20°C and thaw-mounted on an area of 75 mm × 25 mm indium tin oxide glass for MS imaging.

### AP-MALDI-TOF imaging

Brain slices were brought to room temperature in order to be desiccated with nitrogen gas for approximately 25 min. A solution of 25 mg/ml 2,5-dihydroxybenzoic acid in acetonitrile/water at 1:1 (v/v) and 0.5% trifluoroacetic acid was prepared for matrix coating. Longitudinal sections of the mouse brain were coated with a homogeneous layer of matrix using an ultrasonic atomizer prior to MALDI imaging. The samples were stained on indium tin oxide glass and placed vertically under an atomizer. MSI analyses were performed using a Maxis Impact Q-TOF instrument (Bruker, Germany), coupled to a home-built AP-MALDI source instead of an ESI source in this work. A Nd: YAG laser (335 nm, 10 μJ/pulse, 10 ns/pulse) operating at a rate of 1 kHz was used as laser source. The ion images were acquired with a pixel size of 120 μm (“medium” focus setting) and the spectrum of each pixel represented ion signals summed from 500 laser shots. Mass spectra were acquired in reflectron mode over a mass range of m/z 120–1700. Data acquisition and processing were performed using DataAnalysis software (Bruker, Germany, https://www.bruker.com/en/products-and-solutions/mass-spectrometry/ms-software.html). The reconstruction and restoration of imaging data were performed using MATLAB@R2018a software (https://ww2.mathworks.cn/). The spatial content distribution of a specific m/z ion was obtained by peak extraction, normalization, and finally image reconstruction.

### Statistical analysis

Principal components analysis (PCA) and partial least squares discriminant analysis (PLS-DA) were performed using SIMCA-p 16.0. We applied univariate analysis (*t* test) to calculate the statistical significance (*P*-value). The metabolites with VIP > 1 and *P*-value< 0.05 and fold change (FC)≥ 2 or FC≤ 0.5 were considered to be differential metabolites. Volcano plots were used to filter the metabolites of interest which are based on the log2 (FC) and -log10 (*P*-value) of the metabolites. For clustering heat maps, the data were normalized using the z-scores of the intensity areas of the differential metabolites.

Data were analyzed using GraphPad Prism 8.0 (https://www.graphpad-prism.cn/). The data are presented as the arithmetic mean ± SD. Two-sided Student’s t-tests were used to determine significant differences between the groups studied. Statistically significant differences between groups were determined using ANOVA of variance, and Dunnett’s test was used for comparisons among multiple groups. Two-way ANOVA followed by Bonferroni’s post hoc test was used to evaluate multiple comparisons. For all analyses, ∗*P* < 0.05, ∗∗*P* < 0.01, and ∗∗∗*P* < 0.001 were considered statistically significant.

## Results

### Hypobaric hypoxia impaired cognitive behavior in mice

**Fig. 1:**
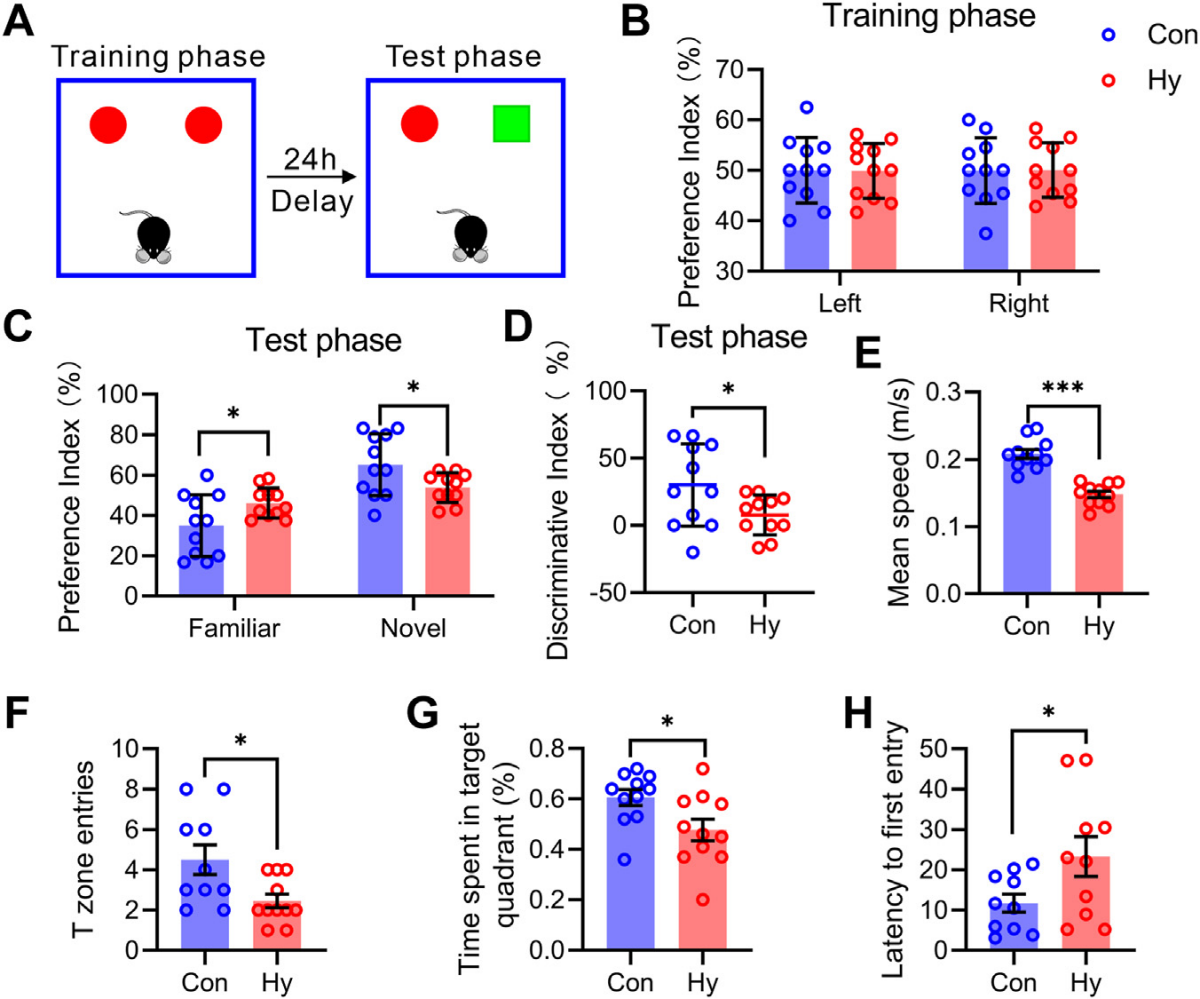
Dysfunctional recognition in hypobaric hypoxia exposed mice. A: Schematic illustration of the strategy for a novel objective recognition test. B–D: Preference and discriminative indices of mice during the (B) training and (C, D) test phases of the novel object recognition test. E–H: (E) Mean swimming, (F) number of T zone entries, (G) time spent in the target quadrant, and (H) escape latency to first entry in the test phase of the Morris water maze. Significance was calculated by Student’s *t* test, and statistically significant changes (∗*P* < 0.05) are marked by asterisks. Data are represented as mean ± standard deviation (n = 11, 11).

We first detected the changes in the cognitive function in mice exposed to hypobaric hypoxia. Neurobehavioral assessments in mice, including new object recognition (NOR) and Morris water maze (MWM) tests, were performed. The mice were exposed to hypobaric hypoxia (7000 m) for 1 day after becoming familiar with the old object, and their preference for the new object was tested. The results showed that hypobaric hypoxia induced cognitive defects manifested as a lower preference for the new object (Fig. 1A–D). In the MWM test, after five days of training and one day of treatment in the decompression chamber, the test phase was performed without an escape platform. The results showed that hypobaric hypoxia stimulation impaired the motor and spatial memory abilities of the mice, as indicated by the slower swimming speed of mice traveling a long distance to the escape platform when compared to the Con group (Fig. 1E–H). These results suggested that exposure to hypobaric hypoxia impairs cognitive function to a certain extent in mice.

### Hypobaric hypoxia induced morphological structure of hippocampus impairment

**Fig. 2:**
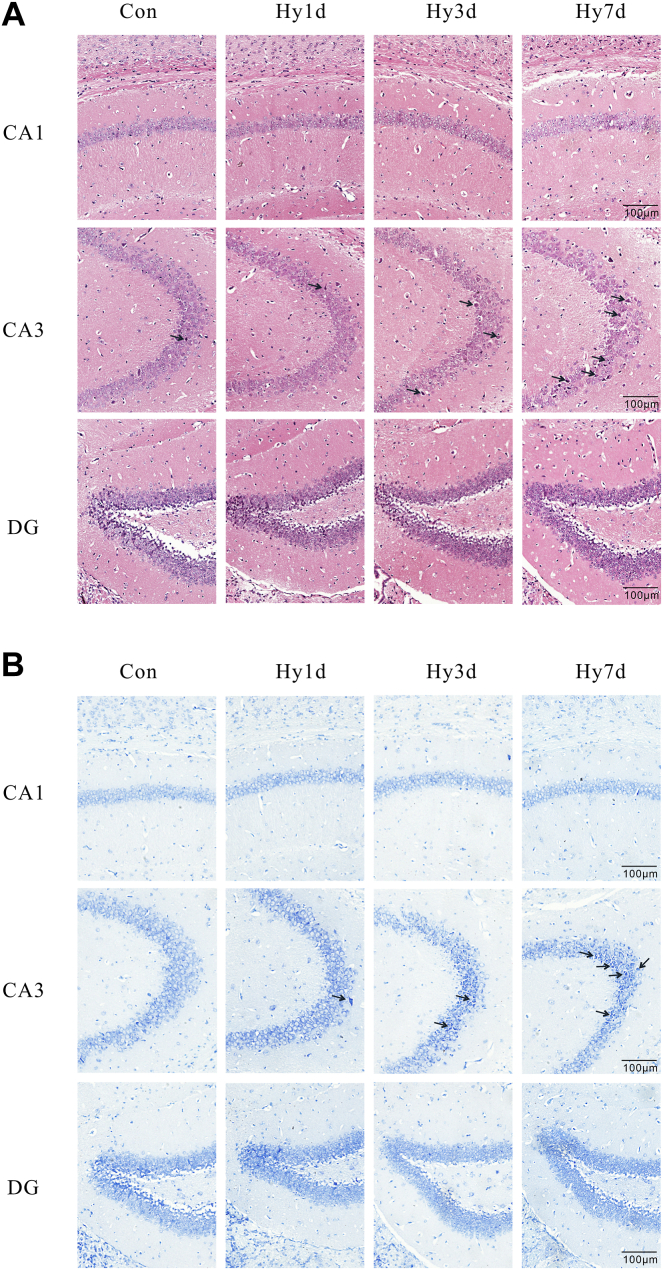
Morphological analysis of hypoxia-induced tissue injury in CA1, CA3, and DG regions. Representative H&E (A) and Nissl staining (B) photomicrographs of hippocampal sections (n = 3). CA, cornu ammonis; DG, dentate gyrus.

In order to explore whether the impaired NOR and spatial memory caused by hypoxia exposure were related to neural damage in the hippocampus, we further observed the morphological changes in the hippocampus with H&E and Nissl staining after exposure to 7000 m hypobaric hypoxia for 1, 3, and 7 days (Hy1d, Hy3d, and Hy7d). Figure 2 shows the representative micrographs of H&E and Nissl staining of the CA1, CA3, and DG regions of the hippocampus for each group. H&E staining indicated normal morphology and a clear boundary of the neurons in the CA3 region of the control (Con) group, while the neurons exhibited abnormal neuronal morphology with shrunken neurons, dark nuclear staining, and even disappearance with prolonged exposure to hypobaric hypoxia, showing loose arrangements and disorganizations (Fig. 2A). Nissl staining was performed to investigate the cytoarchitectural variation in the hippocampus induced by hypobaric hypoxia exposure (Fig. 2B). There was no significant difference seen in the Nissl staining of the CA1 region among the Hy1d, Hy3d, and Hy7d groups. However, neuronal shrinkage and increased cytosolic staining were observed in the CA3 region of Hy1d group. Three days after hypobaric hypoxia, these changes were more noticeable, and the cells were arranged loosely. After 7 days of hypoxic exposure, we found that the neurons were disorganized and also reduced in number, with a dark cytoplasm and small condensed nuclei.

### Global lipidomic profiling of the hippocampus after hypobaric hypoxic exposure

**Fig. 3:**
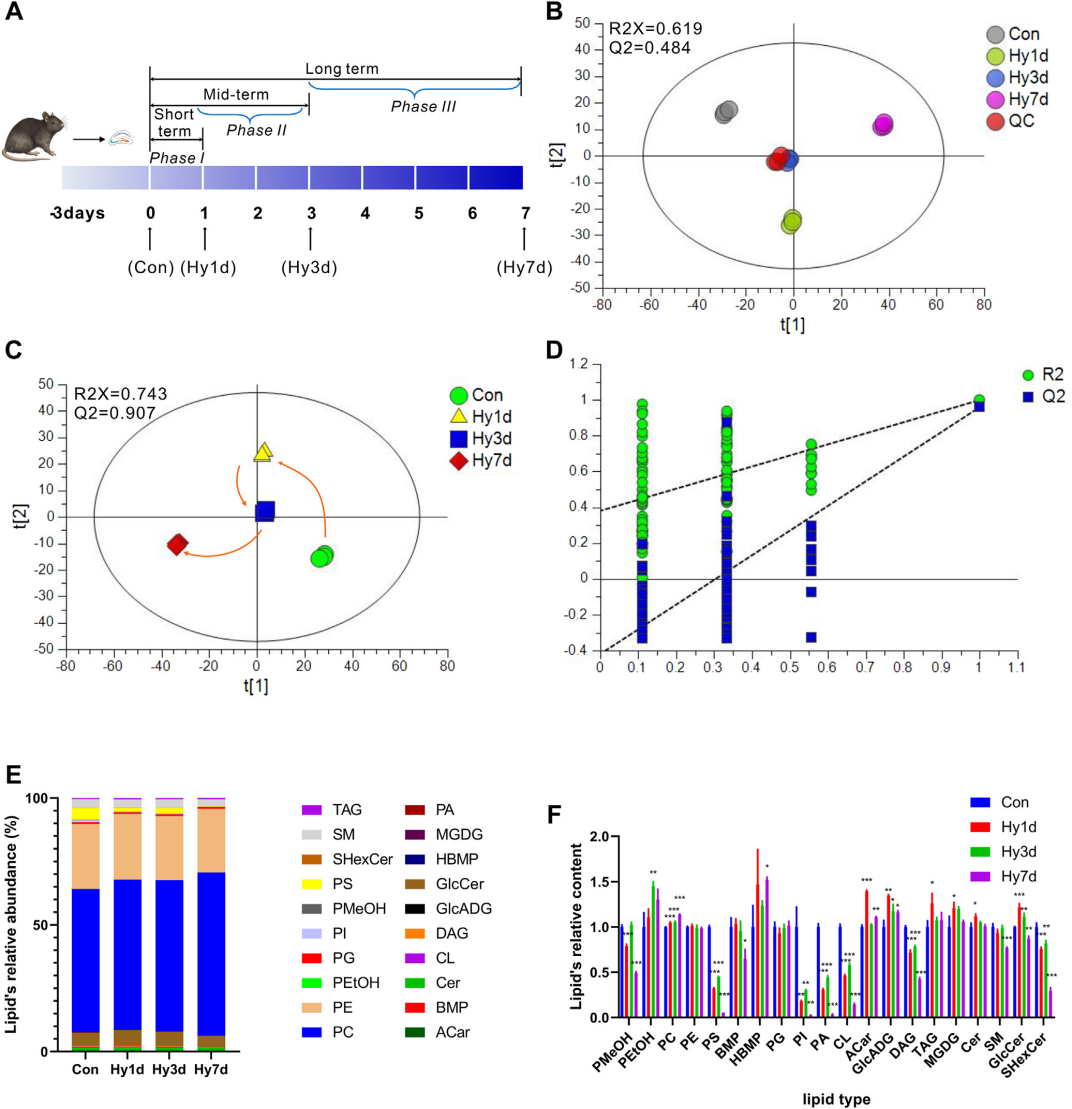
Pattern recognition analysis of mouse brain lipids. A: Graphic illustration to acquire mouse exposed to hypobaric hypoxia. B: Principal component analysis (PCA) of 12 mouse hippocampus lipidome samples and corresponding Quality Control samples (QC, labeled red). QC samples are highly clustered, demonstrating low technical variance and high reliability of lipidomic. C, D: Partial least square discriminant analysis (PLS-DA) of all mouse hippocampus lipidome samples. Samples are colored by different duration of hypoxia exposure. E: Lipid’s relative abundance. F: Lipid’s relative content. Significance was calculated by Student’s *t* test, and statistically significant changes (∗∗∗*P* < 0.001, ∗∗*P* < 0.01, ∗*P* < 0.05) are marked by asterisks. Data are represented as mean ± standard deviation (n = 3).

Since lipids have been shown to play an important role in cognitive function, nontargeted lipidomic analysis of mouse hippocampal tissues was performed in order to explore the possible role of lipid metabolism. A schematic representation of this process is shown in Fig. 3A.

Lipidomics data were collected in positive and negative ion modes. Lipidomics analyses were conducted to decipher the hippocampal lipidome during hypobaric hypoxia exposure (Fig. 3A). Representative total ion chromatograms for each group are shown in supplemental Fig. S1. The acquired data were used to establish PCA model with unit variance scaling. The PCA scores plot (Fig. 3B) showed a clear cluster of the QC samples (R2X = 0.619 and Q2 = 0.484), indicating high stability and also the repeatability of the analysis system. The PCA provided an overview of all the groups; however, the variables responsible for the differences in each cluster remained unclear. PLS-DA model, a supervised pattern recognition, was used in finding differentiated lipids. The score plot of the PLS-DA model revealed an obvious separation between the Con, Hy1d, Hy3d, and Hy7d groups, indicating that lipid alterations progressed gradually along with the hypobaric hypoxia exposure (Fig. 3C). The permutation plot with 200-times test showed that the R2 and Q2 generated from random permutations were higher than the R2 and Q2 distributions of the original PLS-DA model, indicating that the original PLS-DA model was efficient and reliable, with a low risk of overfitting (Fig. 3D). The PLS-DA showed an overall trend of shifting, which indicated that the lipid metabolic processes in the hippocampal region of mice were altered with prolonged exposure to hypobaric hypoxia.

A total of 1142 lipids were identified in positive and negative ion modes, and all the lipids were analyzed by hierarchical clustering to better observe the changes and relationships between lipids in each group. The results showed a correlation between the exposure time of hypobaric hypoxia and lipidic changes (supplemental Fig. S2). Using untargeted lipidomics, these molecular lipid species were annotated, corresponding to 20 major lipid classes in the mouse hippocampus: TAGs, diacylglycerols (DAGs), PSs, phosphatidylcholines (PCs), phosphatidylethanolamines (PEs), PIs, sphingomyelins (SMs), phosphatidic acids (PAs), phosphatidylglycerols (PGs), cardiolipins (CLs), ceramides (Cers), acyl carnitine, hexosylceramide sulfatides, oxidized lipids (phosphatidylmethanol, PMeOH and phosphatidylethanol, PEtOH) and other lipids (Fig. 3E). The level of PC was elevated in Hy1d, Hy3d, and Hy7d, and PC was the most abundant lipid present according to the LC-MS lipidomics analysis (Fig. 3E, F). For phospholipids, the contents of PMeOH, PS, PI, PA, and CL decreased in the Hy1d group. In the Hy3d group, the levels of PMeOH, PS, PI, PA, and CL partially recovered partially; however, in the Hy7d group, the levels of PMeOH, PS, PI, PA, and CL in the hippocampus decreased significantly. We further validated the changes detected by lipidomics by determining the mRNA levels of the enzymes related to lipid metabolism in the mouse hippocampus after 12, 24, and 72 h of hypoxic exposure. In the glycerol 3-phosphate pathway, elevated levels of *Lipin1* and *Dgat2* facilitated TAG synthesis, which may have led to a decrease in DAG and an increase in TAG, consistent with the lipidomics results (supplemental Fig. S3A–C). DAG is the source of synthesis of other phospholipids; when DAG is reduced, other phospholipids are reduced accordingly. During the interconversion of PC, PS, and PE, *Ptdss1* and *Pemt* showed no differences after exposure to hypobaric hypoxia (supplemental Fig. S3D–H). The levels of *Ptdss2* and *Pisd* decreased significantly at 12 and 24 h hours of hypobaric hypoxia exposure and then rebounded after 72 h of hypobaric hypoxia exposure, which is consistent with the lipid changes of hypobaric hypoxia exposure described by the lipidomics results (supplemental Fig. S3D–H). In the biosynthesis of CL, *Cds1*, *Pgs1*, *Cls1* declined the most after 12 h of hypobaric hypoxia exposure, after which their levels rebounded (supplemental Fig. S3I–N). The mRNA level of *Pgs1* even increased after 72 h of hypobaric hypoxia exposure. The mRNA level of *Cdipt*, a key enzyme in the synthesis of PI, was also found to be significantly reduced after 12 h and 24 h of hypobaric hypoxia exposure (supplemental Fig. S3O). The aforementioned results indicated that the PC content was elevated and PMeOH, PS, PI, PA, and CL were reduced in hippocampal tissue exposed to hypobaric hypoxia, and the elevation or reduction worsened with prolonged hypoxic exposure, which was associated with the lipid metabolism-related enzymes including decreased *Ptdss2*, *Pisd, Cds1*, *Pgs1*, *Cls1*, *Cdipt*, and increased *Pgs1*.

### Diversity of molecular lipid species in hippocampus after hypobaric hypoxia exposure

**Fig. 4:**
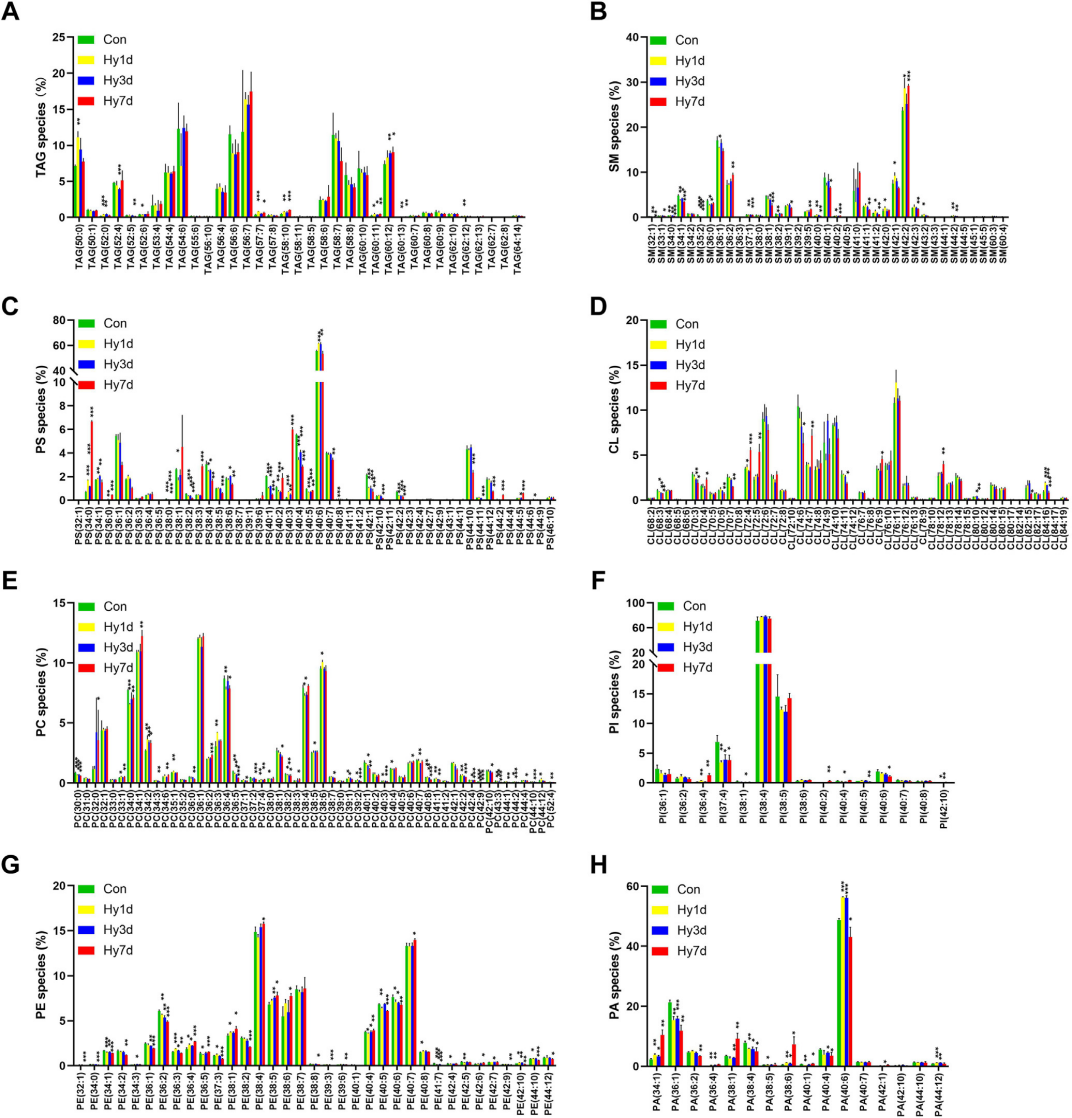
Diverse molecular lipid species of hippocampus in mouse exposed to hypoxia. Lipid profile for (A) TAG, (B) SM, (C) PS, (D) CL, (E) PC, (F) PE, (G) PI, and (H) PA. Significance was calculated by Student’s *t* test, and statistically significant changes (∗∗∗*P* < 0.001, ∗∗*P* < 0.01, ∗*P* < 0.05) are marked by asterisks. Data are represented as mean ± standard deviation (n = 3). CL, cardiolipin; PA, phosphatidic acid; PC, phosphatidylcholine; PE, phosphatidylethanolamines; PI, phosphatidylinositol; PS, phosphatidylserine; SM, sphingomyelin; TAG, triacylglyceride.

Figure 4 shows the profiles of the molecular species of the eight major lipid classes (TAG, SM, PS, CL, PC, PE, PI, and PA) in male mice at different hypobaric hypoxia exposure stages. The abundance of each lipid was normalized to the total abundance of the corresponding lipid class in order to determine the proportion of each molecular lipid species within the lipid class.

TAG 56:7 was the most abundant TAG species in the glyceride class (Fig. 4A). Two types of saturated TAGs, TAG 50:0 and TAG 52:0, were detected. TAG 50:0 accounted for approximately 10% of the total TAG. Both of the TAGs increased in the Hy1d group compared to those in the Con group; subsequently, their contents gradually decreased. The content of some unsaturated TAGs tended to increase gradually, that is TAG 58:10 and TAG 60:12.

In the sphingolipids class, SM 42:2 was a prominent species, accounting for approximately 30% of the total sphingolipids. The monounsaturated SM content showed a certain change trend. The content of most monounsaturated SM decreased on the first day of hypobaric hypoxia, recovered on the third day of hypobaric hypoxia, and then decreased further on the seventh day of hypobaric hypoxia exposure (Fig. 4B).

In the glycerophospholipid class, the predominant PS species in the hippocampal region of mice was polyunsaturated PS 40:6 (Fig. 4C). PS showed a certain regularity with the prolongation of hypobaric hypoxia exposure time. The contents of PS species (PS 34:0, PS 36:0, and PS 38:0) with saturated fatty acids were increased in the Hy1d group and then increased heavily in the Hy7d group, which was not significant for other phospholipids. It is observed that part of PS species containing unsaturated fatty acids (e.g. PS 38:4, PS 40:1, PS 40:4, and PS 42:1) had a lower proportion in Hy1d group and the proportion in Hy7d much lower compared with Con group. CL is a unique, nonbilayer-forming glycerophospholipid present in the mitochondrial membranes. For the CL class, most CL species decreased significantly after 7 days of hypobaric hypoxia exposure (Fig. 4D). However, a portion of the CL (e.g., CL 70:4, CL 72:4, CL 72:5, CL 74:7, and CL78:12) remained abnormally elevated in the Hy7d group, which may be due to cellular respiratory compensation. The CL is an important component of mitochondria and plays an important role in cellular respiration. When exposed to hypobaric hypoxia, the composition of the CL may be regulated in order to stabilize the mitochondrial membrane and thus perform its function. The PC and PE in brain cells often control the membrane anchoring of proteins (26). The prominent of PCs were PC 34:1 and PC 36:1, both of which accounted for approximately 20% of the total PC (Fig. 4E). PC was the most abundant lipid, but its content showed an irregular trend with time of hypobaric hypoxia exposure to hypobaric hypoxia. In the PE class, the majority of the PE species decreased with the prolonged exposure to hypobaric hypoxia, whereas some of the PE species showed an opposite trend (PE 36:4, PE 38:1, PE 38:4, PE 38:5, PE 38:6, and PE 40:7) (Fig. 4G). In the PI class, the greatest abundance was observed for PI 38:4, with no significant differences (Fig. 4F). PI 37:4 and PI 40:6 decreased trend with the hypobaric hypoxic exposure. PA is the simplest cellular glycerophospholipid involved in the regulation and amplification of many cellular signaling pathways and functions, as well as in membrane rearrangements (27). For the PA class, PA 40:6 was prominent species, accounting for over 40% of the total PAs (Fig. 4H). The PA 34:1, PA 38:1, and PA 38:6 contents were significantly elevated in the Hy7d group, whereas the others showed a decreasing trend.

### Changes in the differential lipids with a prolonged hypobaric hypoxia exposure time

**Fig. 5:**
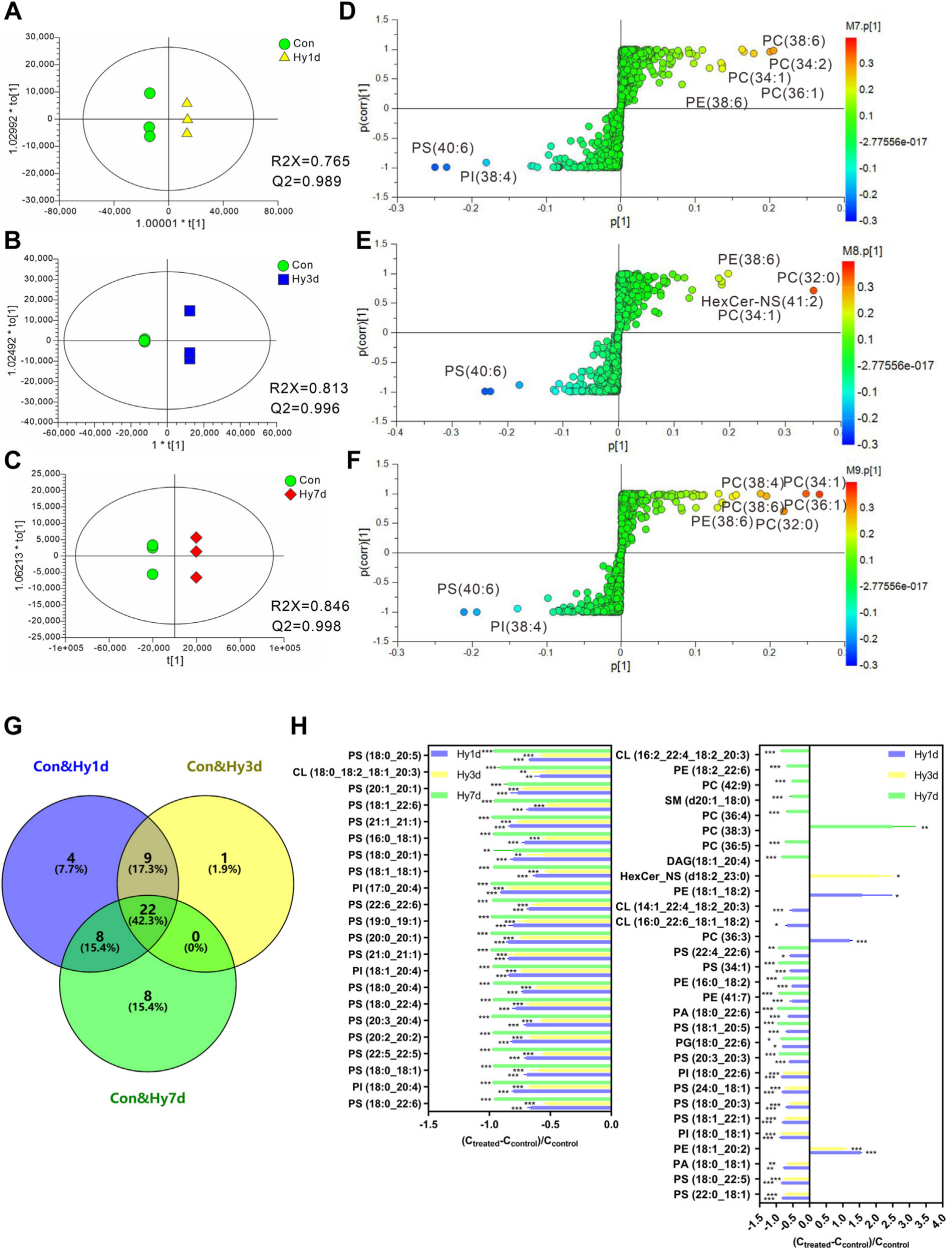
OPLS-DA analysis of the metabolic samples with prolonged exposure time. Panels A–C represents the comparison of the Hy1d, Hy3d, and Hy7d group with the Con group respectively, Panels D–F are S-Plot diagrams obtained by comparing Hy1d, Hy3d, and Hy7d respectively. G: Venn diagram of the differential metabolites compared between the treatment and con group at different exposure time. H: Specific differential metabolites and their concentration variation between the experimental group and the Con group. The X-axis represents the difference of metabolites between the treatment group and the Con group, and the concentration difference was expressed as (C_treated_ − C_control_)/C_control_. Significance was calculated by Student’s *t* test, and statistically significant changes (∗∗∗*P* < 0.001, ∗∗*P* < 0.01, ∗*P* < 0.05) are marked by asterisks. Data are represented as mean ± standard deviation (n = 3). OPLS-DA, orthogonal partial least squares discriminant analysis.

We remodeled the data to investigate the effect of hypobaric hypoxia on the hippocampal region of the brain lipid metabolism in mice with prolonged exposure time (Fig. 5A–C). Orthogonal PLS-DA was performed on pairwise lipid data consisting of Con-Hy1d, Con-Hy3d, and Con-Hy7d. None of the three orthogonal PLS-DA models appeared to overfit, confirming that the models were effective. The S-plot method was used for each group to screen for differential metabolites with prolonged exposure times (Fig. 5D–F). The S-plot is a scatter plot resulting from a projection-based model that visualizes the variable influence of the model (28). The abscissa of the S-plot represents the load of each substance on the first principal component, and the ordinate represents the correlation coefficient between each substance and the first principal component.

Ultimately, 43, 32, and 38 significantly altered lipids were screened using the criteria of VIP > 1.0, *P* < 0.05 and FC > 2 or FC < 0.5 in the Hy1d, Hy3d, and Hy7d groups compared to the Con group (Fig. 5G, supplemental Figs. S4–S6, Tables 1, 2, and 3, and supplemental Table S2). Among these metabolites, 22 lipids were consistently decreased in all three models (Fig. 5G). As shown in Fig. 5H, the lipids that changed continuously included PS, PI, and CL in all of the three groups studied, of which PS accounted for 18 of the 22 lipids. Lipids were found to change significantly in the Hy1d and Hy3d groups, but not in the Hy7d group, which we considered to be sensitive compounds for the immediate response to hypoxic exposure. For example, PE 18:1-20:2 increased in the Hy1d and Hy3d groups, and the degree of increase in the Hy3d group was lower than that in the Hy1d group. In general, by analyzing the amount of lipids in groups with different exposure times, it can be concluded that the degree of brain metabolism disturbance is strongly linked to the exposure time.

**Table 1:** The differential metabolites between Con and Hy1d group

No.	Lipid name	VIP	*P*_Value	FC	Mean _Con	Mean _Hy1d
1	PS (20:3_20:3)	8.41	9.94E-07	0.39	4.72±0.09E+10	1.82±0.05E+10
2	PS (18:0_22:6)	7.88	6.27E-06	0.34	3.85±0.11E+10	1.30±0.09E+10
3	PI (18:0_20:4)	6.11	1.00E-02	0.20	2.08±0.63E+10	4.10±0.25E+09
4	PC (36:3)	5.50	1.32E-05	2.22	1.02±0.04E+10	2.26±0.07E+10
5	PS (18:0_18:1)	3.76	1.37E-06	0.31	8.38±0.10E+09	2.57±0.20E+09
6	PS (22:5_22:5)	3.05	1.87E-05	0.30	5.49±0.27E+09	1.66±0.09E+09
7	PS (20:2_20:2)	2.92	1.09E-06	0.19	4.30±0.11E+09	8.05±0.57E+08
8	PS (20:3_20:4)	2.86	4.63E-06	0.30	4.79±0.16E+09	1.43±0.07E+09
9	PS (18:0_22:4)	2.84	3.01E-05	0.22	4.24±0.25E+09	9.20±1.16E+08
10	PS (18:0_20:4)	2.81	1.73E-04	0.27	4.51±0.42E+09	1.23±0.04E+09
11	PI (18:1_20:4)	2.79	1.63E-05	0.16	3.83±0.22E+09	6.21±0.41E+08
12	PG(18:0_22:6)	2.54	3.15E-02	0.18	3.81±1.66E+09	7.00±0.19E+08
13	PS (42:1)	2.39	1.35E-05	0.16	2.79±0.16E+09	4.42±0.23E+08
14	PS (20:0_20:1)	2.18	1.04E-05	0.16	2.30±0.12E+09	3.57±0.36E+08
15	PS (38:1)	2.14	2.67E-05	0.20	2.35±0.12E+09	4.60±0.91E+08
16	PS (22:6_22:6)	2.10	1.65E-04	0.32	2.69±0.23E+09	8.70±0.42E+08
17	PI (17:0_20:4)	2.07	9.97E-06	0.09	1.94±0.11E+09	1.83±0.08E+08
18	PS (18:1_18:1)	2.03	1.81E-06	0.38	2.71±0.05E+09	1.02±0.05E+09
19	PS (18:1_20:5)	1.97	6.19E-06	0.30	2.28±0.08E+09	6.90±0.45E+08
20	PS (18:0_20:1)	1.76	8.82E-07	0.19	1.58±0.03E+09	3.06±0.32E+08
21	PA (18:0_22:6)	1.69	2.59E-05	0.36	1.83±0.09E+09	6.56±0.26E+08
22	PE (41:7)	1.65	8.80E-04	0.48	2.18±0.15E+09	1.04±0.17E+09
23	PS (16:0_18:1)	1.60	1.25E-06	0.29	1.47±0.03E+09	4.26±0.28E+08
24	PS (42:2)	1.51	1.36E-05	0.17	1.13±0.06E+09	1.91±0.14E+08
25	PE (16:0_18:2)	1.48	4.93E-07	0.47	1.71±0.01E+09	8.08±0.23E+08
26	PS (18:1_22:6)	1.45	6.04E-05	0.32	1.27±0.08E+09	4.08±0.21E+08
27	PS (20:1_20:1)	1.44	8.34E-05	0.23	1.12±0.06E+09	2.59±0.65E+08
28	CL (18:0_18:2_18:1_20:3)	1.36	3.61E-03	0.42	1.37±0.22E+09	5.70±0.62E+08
29	PS (18:0_20:5)	1.36	5.08E-07	0.33	1.13±0.02E+09	3.71±0.04E+08
30	PS (22:0_18:1)	1.31	7.43E-05	0.18	8.65±0.71E+08	1.58±0.19E+08
31	PS (34:1)	1.29	1.57E-05	0.42	1.18±0.04E+09	5.01±0.24E+08
32	PS (18:0_22:5)	1.27	9.42E-07	0.16	7.79±0.22E+08	1.22±0.04E+08
33	PA (18:0_18:1)	1.23	3.80E-05	0.22	8.03±0.53E+08	1.80±0.12E+08
34	PS (22:4_22:6)	1.23	1.84E-02	0.41	1.18±0.31E+09	4.89±0.11E+08
35	PE (18:1_20:2)	1.19	5.64E-06	2.54	3.76±0.30E+08	9.53±0.09E+08
36	PI (18:0_18:1)	1.17	3.03E-03	0.13	6.70±1.57E+08	8.49±1.92E+07
37	CL (16:0_22:6_18:1_18:2)	1.11	4.11E-02	0.33	9.11±3.55E+08	2.97±0.46E+08
38	CL (14:1_22:4_18:2_20:3)	1.07	4.34E-04	0.48	9.11±0.42E+08	4.35±0.65E+08
39	PS (18:1_22:1)	1.06	2.40E-05	0.20	5.82±0.32E+08	1.18±0.17E+08
40	PS (18:0_20:3)	1.05	3.49E-04	0.32	6.70±0.66E+08	2.13±0.24E+08
41	PE (18:1_18:2)	1.04	4.65E-02	2.58	3.44±1.17E+08	8.86±3.08E+08
42	PS (24:0_18:1)	1.01	2.45E-07	0.18	5.10±0.07E+08	9.15±0.76E+07
43	PI (18:0_22:6)	1.01	8.98E-06	0.16	4.96±0.25E+08	7.91±0.53E+07

**Table 2:** The differential metabolites between Con and Hy3d group

No.	Lipid name	VIP	*P*_Value	FC	Mean _Con	Mean _Hy3d
1	PS (18:0_22:6)	7.78	1.80E-05	0.47	3.85±0.11E+10	1.80±0.10E+10
2	HexCer_NS (d18:2_23:0)	6.29	4.90E-02	3.05	7.98±9.65E+09	2.44±0.31E+10
3	PI (18:0_20:4)	6.03	1.89E-02	0.33	2.08±0.63E+10	6.94±0.28E+09
4	PS (18:0_18:1)	3.82	1.41E-04	0.41	8.38±0.10E+09	3.42±0.60E+09
5	PS (22:5_22:5)	2.99	5.58E-05	0.45	5.49±0.27E+09	2.45±0.12E+09
6	PS (18:0_22:4)	2.90	4.98E-05	0.33	4.24±0.25E+09	1.38±0.10E+09
7	PS (20:2_20:2)	2.89	3.73E-06	0.35	4.30±0.11E+09	1.49±0.08E+09
8	PI (18:1_20:4)	2.88	2.97E-05	0.27	3.83±0.22E+09	1.01±0.06E+09
9	PS (20:3_20:4)	2.85	8.48E-06	0.43	4.79±0.16E+09	2.05±0.04E+09
10	PS (18:0_20:4)	2.84	3.45E-04	0.39	4.51±0.42E+09	1.75±0.05E+09
11	PS (42:1)	2.56	4.50E-05	0.20	2.79±0.16E+09	5.70±1.29E+08
12	PS (20:0_20:1)	2.24	1.64E-05	0.26	2.30±0.12E+09	5.97±0.28E+08
13	PS (38:1)	2.20	9.65E-05	0.30	2.35±0.12E+09	7.00±1.37E+08
14	PS (22:6_22:6)	2.20	3.36E-04	0.39	2.69±0.23E+09	1.04±0.11E+09
15	PI (17:0_20:4)	2.17	2.90E-05	0.18	1.94±0.11E+09	3.48±0.70E+08
16	PS (18:1_18:1)	2.06	1.91E-06	0.47	2.71±0.05E+09	1.28±0.04E+09
17	PS (18:0_20:1)	1.60	1.72E-03	0.44	1.58±0.03E+09	6.93±2.04E+08
18	PS (42:2)	1.59	2.20E-05	0.24	1.13±0.06E+09	2.71±0.21E+08
19	PS (20:1_20:1)	1.54	4.01E-05	0.28	1.12±0.06E+09	3.17±0.30E+08
20	PS (16:0_18:1)	1.52	1.71E-05	0.47	1.47±0.03E+09	6.93±0.49E+08
21	CL (18:0_18:2_18:1_20:3)	1.42	8.91E-03	0.46	1.37±0.22E+09	6.36±1.55E+08
22	PS (18:1_22:6)	1.40	1.56E-04	0.48	1.27±0.08E+09	6.05±0.13E+08
23	PS (18:0_20:5)	1.38	3.44E-06	0.44	1.13±0.02E+09	4.94±0.21E+08
24	PS (22:0_18:1)	1.36	1.17E-04	0.28	8.65±0.71E+08	2.41±0.15E+08
25	PS (18:0_22:5)	1.32	2.38E-06	0.24	7.79±0.22E+08	1.91±0.13E+08
26	PA (18:0_18:1)	1.26	6.25E-05	0.33	8.03±0.53E+08	2.66±0.02E+08
27	PI (18:0_18:1)	1.25	3.87E-03	0.18	6.70±1.57E+08	1.19±0.26E+08
28	PS (18:1_22:1)	1.11	3.93E-05	0.29	5.82±0.32E+08	1.67±0.18E+08
29	PE (18:1_20:2)	1.07	1.40E-04	2.04	3.76±0.30E+08	7.65±0.37E+08
30	PS (18:0_20:3)	1.06	5.99E-04	0.42	6.70±0.66E+08	2.80±0.20E+08
31	PI (18:0_22:6)	1.06	2.00E-05	0.24	4.96±0.25E+08	1.17±0.13E+08
32	PS (24:0_18:1)	1.04	2.48E-07	0.28	5.10±0.07E+08	1.44±0.06E+08

**Table 3:** The differential metabolites between Con and Hy7d group

No.	Lipid name	VIP	*P*_Value	FC	Mean _Con	Mean _Hy7d
1	PS (20:3_20:3)	7.12	1.10E-07	0.07	4.72±0.09E+10	3.45±0.02E+09
2	PS (18:0_22:6)	6.54	5.60E-07	0.04	3.85±0.11E+10	1.63±0.22E+09
3	PI (18:0_20:4)	4.69	5.11E-03	0.03	2.08±0.63E+10	6.08±0.84E+08
4	PS (18:0_18:1)	3.07	1.57E-08	0.03	8.38±0.10E+09	2.82±0.25E+08
5	DAG(18:1_20:4)	2.67	1.69E-07	0.16	7.30±0.13E+09	1.17±0.05E+09
6	PC (36:5)	2.61	2.16E-05	0.27	8.09±0.43E+09	2.22±0.13E+09
7	PS (22:5_22:5)	2.49	4.15E-06	0.03	5.49±0.27E+09	1.51±0.21E+08
8	PS (20:3_20:4)	2.33	9.04E-07	0.02	4.79±0.16E+09	9.91±1.30E+07
9	PS (18:0_20:4)	2.24	5.53E-05	0.03	4.51±0.42E+09	1.51±0.13E+08
10	PS (20:2_20:2)	2.20	3.42E-07	0.03	4.30±0.11E+09	1.31±0.10E+08
11	PS (18:0_22:4)	2.18	8.80E-06	0.03	4.24±0.25E+09	1.41±0.06E+08
12	PI (18:1_20:4)	2.07	8.52E-06	0.03	3.83±0.22E+09	1.13±0.08E+08
13	PC (38:3)	1.98	2.96E-03	3.51	1.41±0.15E+09	4.95±0.94E+09
14	PG(18:0_22:6)	1.81	2.71E-02	0.14	3.81±1.66E+09	5.45±0.46E+08
15	PS (42:1)	1.78	6.99E-06	0.02	2.79±0.16E+09	4.26±1.25E+07
16	PC (36:4)	1.75	1.53E-05	0.32	3.93±0.16E+09	1.27±0.09E+09
17	SM (d20:1_18:0)	1.75	4.71E-04	0.48	5.16±0.09E+09	2.47±0.44E+09
18	PS (18:1_18:1)	1.74	6.83E-08	0.04	2.71±0.05E+09	9.66±0.99E+07
19	PS (22:6_22:6)	1.74	3.67E-05	0.03	2.69±0.23E+09	7.05±0.53E+07
20	PS (38:1)	1.64	4.86E-06	0.02	2.35±0.12E+09	4.00±0.28E+07
21	PS (20:0_20:1)	1.62	4.69E-06	0.01	2.30±0.12E+09	3.24±0.51E+07
22	PS (18:1_20:5)	1.59	9.87E-07	0.04	2.28±0.08E+09	1.02±0.11E+08
23	PE (41:7)	1.52	1.91E-05	0.08	2.18±0.15E+09	1.74±0.14E+08
24	PI (17:0_20:4)	1.49	7.11E-06	0.02	1.94±0.11E+09	3.04±0.33E+07
25	PA (18:0_22:6)	1.43	4.46E-06	0.03	1.83±0.09E+09	6.35±0.88E+07
26	PS (16:0_18:1)	1.28	9.91E-08	0.04	1.47±0.03E+09	5.44±0.83E+07
27	PE (16:0_18:2)	1.25	2.20E-07	0.21	1.71±0.01E+09	3.57±0.30E+08
28	PS (18:0_20:1)	1.20	1.05E-03	0.20	1.58±0.03E+09	3.15±2.56E+08
29	CL (18:0_18:2_18:1_20:3)	1.19	6.24E-04	0.09	1.37±0.22E+09	1.25±0.38E+08
30	PS (18:1_22:6)	1.19	1.37E-05	0.04	1.27±0.08E+09	5.22±0.47E+07
31	PC (42:9)	1.18	4.32E-07	0.48	2.31±0.02E+09	1.11±0.03E+09
32	PS (42:2)	1.13	6.70E-06	0.03	1.13±0.06E+09	2.85±0.71E+07
33	PS (18:0_20:5)	1.13	1.32E-07	0.04	1.13±0.02E+09	4.05±0.63E+07
34	PS (34:1)	1.13	1.65E-06	0.08	1.18±0.04E+09	9.12±1.35E+07
35	PE (18:2_22:6)	1.12	2.56E-05	0.32	1.59±0.08E+09	5.08±0.15E+08
36	PS (22:4_22:6)	1.11	3.52E-03	0.06	1.18±0.31E+09	7.14±0.53E+07
37	CL (16:2_22:4_18:2_20:3)	1.08	2.64E-04	0.16	1.20±0.14E+09	1.88±0.24E+08
38	PS (20:1_20:1)	1.05	2.42E-05	0.15	1.12±0.06E+09	1.70±0.37E+08

Based on the results of the time-effect analysis, we conducted a comparative analysis between the Con and Hy1d groups. Hierarchical cluster analysis (supplemental Fig. S7A) revealed that the differential metabolites between the two groups were clearly separated. When compared with the Con group, the brain metabolism of mice was significantly affected after only one extra day of hypobaric hypoxia exposure. In the Hy3d group, the lipid content recovered to some extent, whereas with prolonged exposure time, the lipid content changed significantly after 7-days exposure time (supplemental Fig. S7B). In the correlation coefficient graph (supplemental Fig. S8), it can be seen that PS, PI, CL, and SM are positively correlated with each other and have the same trend of change, whereas they are negatively correlated with PC and PE and have the opposite trend of change, whether they are exposed to hypoxia for 1 or 7 days.

### Localization of lipids in mouse brain section by AP-MALDI MSI

**Fig. 6:**
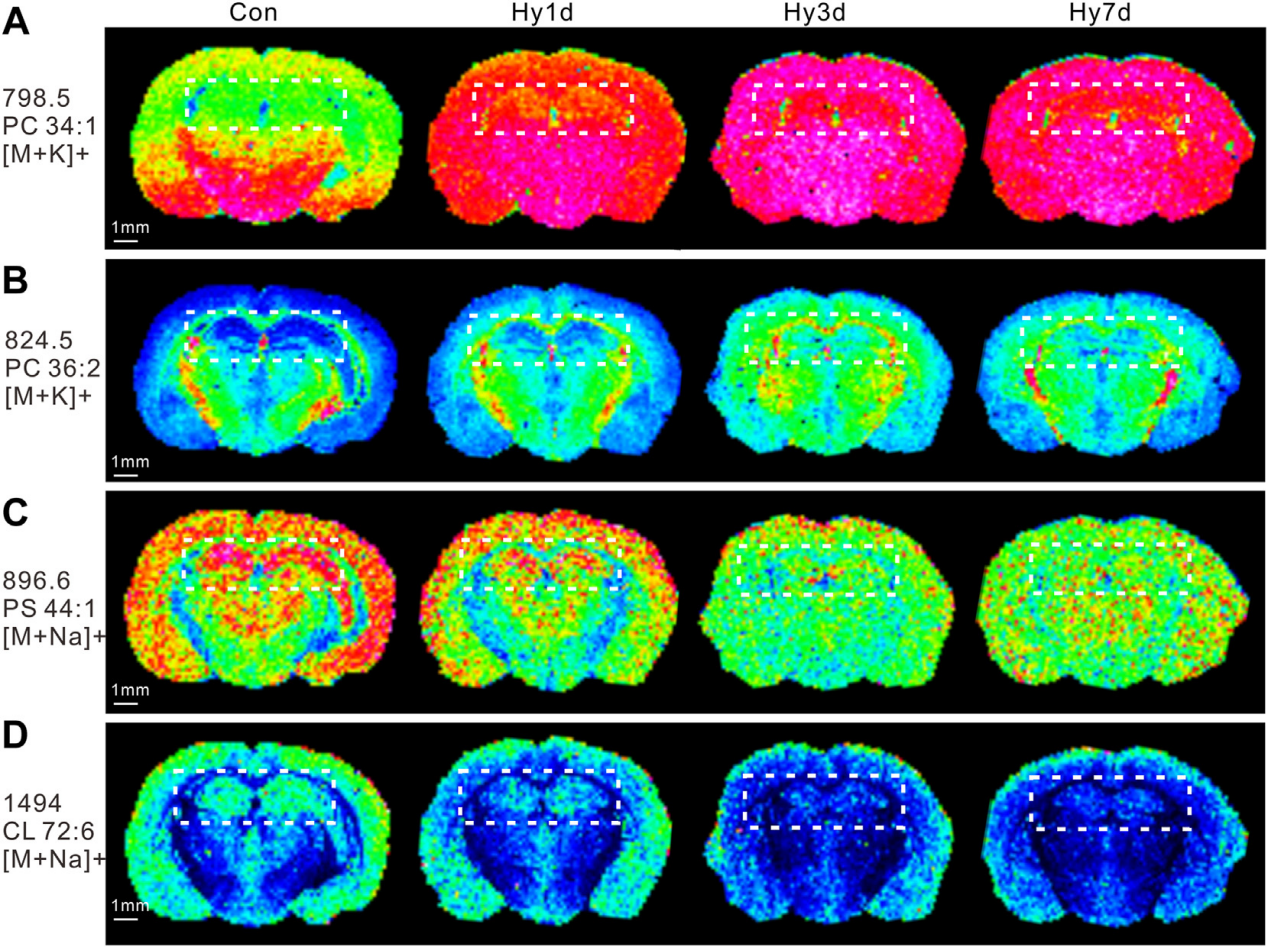
Localization of lipids in whole-brain section by AP-MALDI MSI. The box area circled is the hippocampus. A: Ion image of potassium adduct PC 34:1. B: Ion image of potassium adduct PC 36:2. C: Ion image of sodium adduct of PS 44:1. D: Ion image of sodium adduct of CL 72:6. CL, cardiolipin; MSI, MS imaging; PC, phosphatidylcholine; PS, phosphatidylserine.

Using the rich molecular lipid species information obtained by LC-MS lipidomics, MSI was performed to map the lipids in situ and reveal their spatial localization in the brains. Coronal sections of mouse brains were mapped using AP-MALDI MSI to examine the global distribution of the lipid species. In the positive ion mode, AP-MALDI MS ion images of the main PC lipids, PC 34:1 and PC 36:2, are shown in Fig. 6A, B. The ions at *m/z* 798.5, which are the characteristic fragments of PC lipids, were abundant in the brain (Fig. 6A). In the Con group, PC 34:1 was abundant in the amygdala and hypothalamus of mice, but low in the hippocampus. However, when exposed to hypobaric hypoxia, PC 34:1 increased in Hy1d group in hippocampus of mice, and with the prolongation of hypobaric hypoxia exposure, the level of PC increased even more in the Hy7d group, which was consistent with the lipidomic results (Fig. 6A). Another PC species with the ions at *m/z* 824.5, characterized as PC 36:2, showed a similar trend of content changes in the hippocampus (Fig. 6B). In the lipidomic results, we found a significant decrease in PS content under hypobaric hypoxia exposure, which was consistent with the MSI results. The ions at *m/z* 896.6, characterized as PS 44:1, gradually decreased with prolonged exposure to hypobaric hypoxia in the hippocampus; this decrease was evident in the cortex (Fig. 6C). Similarly, the ions at *m/z* 734.5, CL 72:6, the content of which in the hippocampus decreased significantly with time of hypobaric hypoxia exposure and decreased in the cortex, as shown in Fig. 6D. MSI not only verified the hippocampal lipidomic results, but it also enabled the observation of lipid information in other regions of the brain.

## Discussion

The physiological changes induced by hypoxic exposure are dynamic and complex. Lipids provide the structural basis for cell membranes and are involved in a wide variety of vital tasks in the brain, including signal transduction (29). Various studies have shown that low oxygen exposure can lead to cognitive decline (9, 30, 31). We found that after one day of hypoxic exposure, mice had a reduced ability to discriminate new objects in the NOR experiment and searching for the platform in the MWM experiment, and the morphological results showed neuronal damage in the hippocampal region. The hypobaric hypoxia’s contribution to the brain lipidome has not yet been comprehensively investigated. In this study, we have applied a lipidome strategy based on LC-MS and mass imaging technology with the aim of providing a resource that describes the molecular signature at the lipidome level for dynamic changes in the mouse hippocampus during short-term (1∼7 days) hypoxia exposure (7000 m), explored the metabolic characteristics, and screened for biomarkers. These biomarkers can potentially be used to classify and also predict the metabolic changes over an additional 7-day period of prolonged hypobaric hypoxia exposure. Data analysis revealed that differences in lipid metabolism were almost entirely found along the hypobaric hypoxia exposure-time axis in the brain. These lipidomic differences were consistent with previously published functional and molecular phenotypes (32, 33) that highlighted large lipid differences in the brain during the hypobaric hypoxia exposure, which was also validated by the concordance between metabolite abundances imaging and mRNA levels. Altered lipids in the brain may be responsible for impaired brain functions previously reported in hypoxic brains (34), such as a significant change in spatial reference memory after exposure to hypobaric hypoxia (6100 m) in rats (10, 35).

Several previous studies have shown that hypoxia causes cognitive dysfunction (7, 36). According to a study of 20 male soldiers, exposure to high altitude at 4700 m for 5–7 h adversely affects the mental performances, causing acute mountain sickness (37). Furthermore, Cavaletti *et al.* in their study reported a significant impairment in spatial memory performance in climbers has been reported at altitudes greater than 5000 m (38). Nelson *et al.* found learning impairments in an intact retrieval function in a group of mountaineers returning to sea level at an altitude of 6000 m (31). When ascending to an altitude of 7000 m, cognitive functions, including learning and memory retrieval, are further impaired (30), which is an important attribute of the hippocampus (39). Previous MRI studies of high-altitude sojourners showed gray matter loss and atrophy in several brain regions, along with shrinkage in the hippocampus, indicating neuronal damage upon exposure to high altitudes (40). Similar to our findings, cognitive function appeared to be impaired in mice in the MWM and NOR experiments.

Lipid homeostasis is crucial for organism development, maintenance, and reproduction of the organism (41, 42). However, this homeostasis can be disrupted when an organism is threatened by environmental stress. Growing evidence has shown that membrane lipids played crucial roles in behavioral pathogenesis and neuronal function (43, 44). Lipids, the basic building blocks of membrane structure, have many proteins and peptides embedded in lipid membranes, regulating neuronal structures and functions. Alternatively, lipids can also act as second messengers to mediate signal transduction, thereby influencing behavior (45). Previous preclinical studies have suggested that in cases of cognitive impairment, such as in the neurodegenerative disease Alzheimer’s disease, there are abnormal fluctuations in plasma lipids, such as a decrease in CL levels and an increase in oxidized CL (46). Lipid profiling in our study revealed multiple lipid variations in the hippocampus after exposure to hypobaric hypoxia and showed regular changes with exposure time.

Sphingolipids, especially SM, help in brain myelination surrounding the axons and are thereby directly involved in signal transduction in the brain, which is closely associated with cognitive maturation (47). Myelin sheaths encapsulated on axons reduce the capacitance of the axonal membrane, thus allowing the rapid conduction of action potentials (47). We observed that SM decreased significantly in the hippocampus of the Hy7d group compared to the Con group, with most monounsaturated sphingolipids being significantly reduced. The reduction in myelin caused by the reduced SM under hypoxic exposure increases the membrane capacitance, which could in turn generate a slower signal conduction speed. In addition, SM can be hydrolyzed to Cers, which have multiple functions. Cers may induce apoptosis and contribute to synaptic signaling by promoting budding and vesiculation (48). Therefore, reduced sphingolipid levels may be detrimental for signal transduction.

Glycerophospholipids, which are the main components of cell membranes, are affected during hypoxia exposure. Properties of the neural membrane, such as stability, permeability, and fluidity, are affected when the glycerophospholipids composition is altered, which can even cause neurological disorders (26). PS and PI can determine the surface changes of the membrane, which is important for targeting positively charged proteins (49, 50). The neuronal glycerolipid biosynthesis pathway plays a critical role in the response to injury and axon regeneration (32). Axotomy elevates Lpin1 in retinal ganglion cells, which diverts lipids to the TAG storage lipids synthesis pathway rather than the phospholipid membrane lipids in neurons, thus limiting axon regeneration. We found that *Lpin1* and *Dgat2* were upregulated when exposed to hypoxia for 1 day, resulting in elevated TAG levels and a general decrease in phospholipid levels. When this homeostasis is disrupted by hypoxic exposure, alterations in lipid metabolism potentially leading to limitations in neuronal axon generation, further affecting their cognitive abilities.

Our results demonstrated that exposure to hypobaric hypoxia impaired cognitive function to some extent in mice, induced dramatic variations in hippocampal morphology, and promoted disturbances in lipid metabolism. We proposed a hypoxia-induced pathway network to elucidate the neuronal regulation related to lipid metabolism and localization. This study has suggested that hypobaric hypoxia exposure could lead to the cognitive disorders in a mouse model, which are closely related to lipid changes under hypoxic exposure. Lipidomics is an innovative and promising method for elucidating the potential mechanisms of hypoxia-induced brain damage. We have established a time-response relationship between hypoxia and the lipid levels in the mouse hippocampus, providing new insights into the mechanisms of cognitive impairment induced by hypoxia. This study provides a foundation for future drug interventions to prevent hypoxia-induced brain damage.

## Limitations of the Study

All biochemical findings are based on mice model. Underlying molecular mechanisms remain inadequately understood. We should be very careful when extrapolating findings from rodent animal models to humans.

## Data availability

All data supporting this study are included in the article and supplemental data.

## Supplemental data

This article contains supplemental data.

## Conflict of interest

The author declares that they have no conflicts of interest with the contents of this article.
