# Supplementary material for: Lipidomics and mass spectrometry imaging unveil alterations in mice hippocampus lipid composition exposed to hypoxia
Source: J Lipid Res. 2024 Jun 10;65(7):100575. doi: 10.1016/j.jlr.2024.100575 (PMC11333011; doi:10.1016/j.jlr.2024.100575)
Supplement: Supplemental information [file mmc1.docx]

**Lipidomics and Mass Spectrometry Imaging unveil Alterations in Mice Hippocampus Lipid Composition exposed to Hypoxia**

Jiayue Gao^1^, Zhiying Guo^2^, Ming Zhao^1^, Xiang Cheng^1^, Xiufang Jiang^1^, Yikun Liu^3^, Wenpeng Zhang^3^, Xiangpei Yue^1^, Xuechao Fei^1^, Yaqun Jiang^1^, Lu Chen^1^, Shaojie Zhang^4^, Tong Zhao^1^, Lingling Zhu^1, 5, *^

^1^ Department of Brain Plasticity, Beijing Institute of Basic Medical Sciences, Beijing, 100850, China

^2^ Hepato-pancreato-biliary Center, Beijing Tsinghua Changgung Hospital, Tsinghua University, Beijing, 102218, China

^3^ State Key Laboratory of Precision Measurement Technology and Instruments, Department of Precision Instrument, Tsinghua University, Beijing, 100084, China

^4^ Department of Gastroenterology, the Second Medical Center & National Clinical Research Center for Geriatric Diseases, Chinese PLA General Hospital, Beijing, 100853, China

^5^ Co-Innovation Center of Neuroregeneration, Nantong University, Nantong, 226019, China

***The Corresponding Authors:**

Lingling Zhu, M.D, Ph.D.

Tel: 8610-66931315

Email: linglingzhuamms@126.com

Address: No.27, Taiping Road, Haidian District, Beijing, 100850, China.

**Short title:** Hypobaric Hypoxia alters Lipid Profile of Mice Hippocampus

**Funding sources:** This work was supported by the National Natural Science Foundation of China (grant number: 82072104).


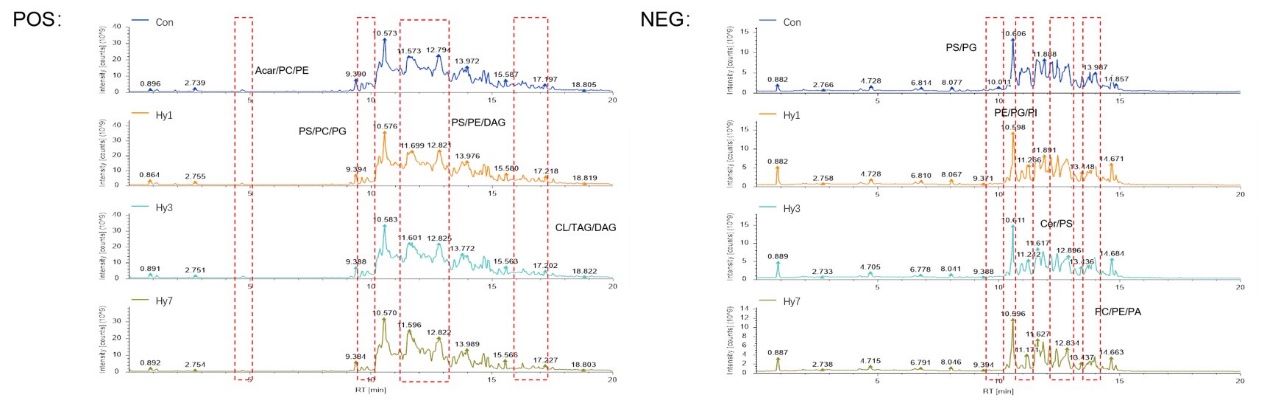


**Figure S1.** Total ion chromatograms (TIC) of lipid extracts from mouse hippocampal tissue in positive and negative ion modes after 1, 3 and 7 days of hypobaric hypoxia exposure.


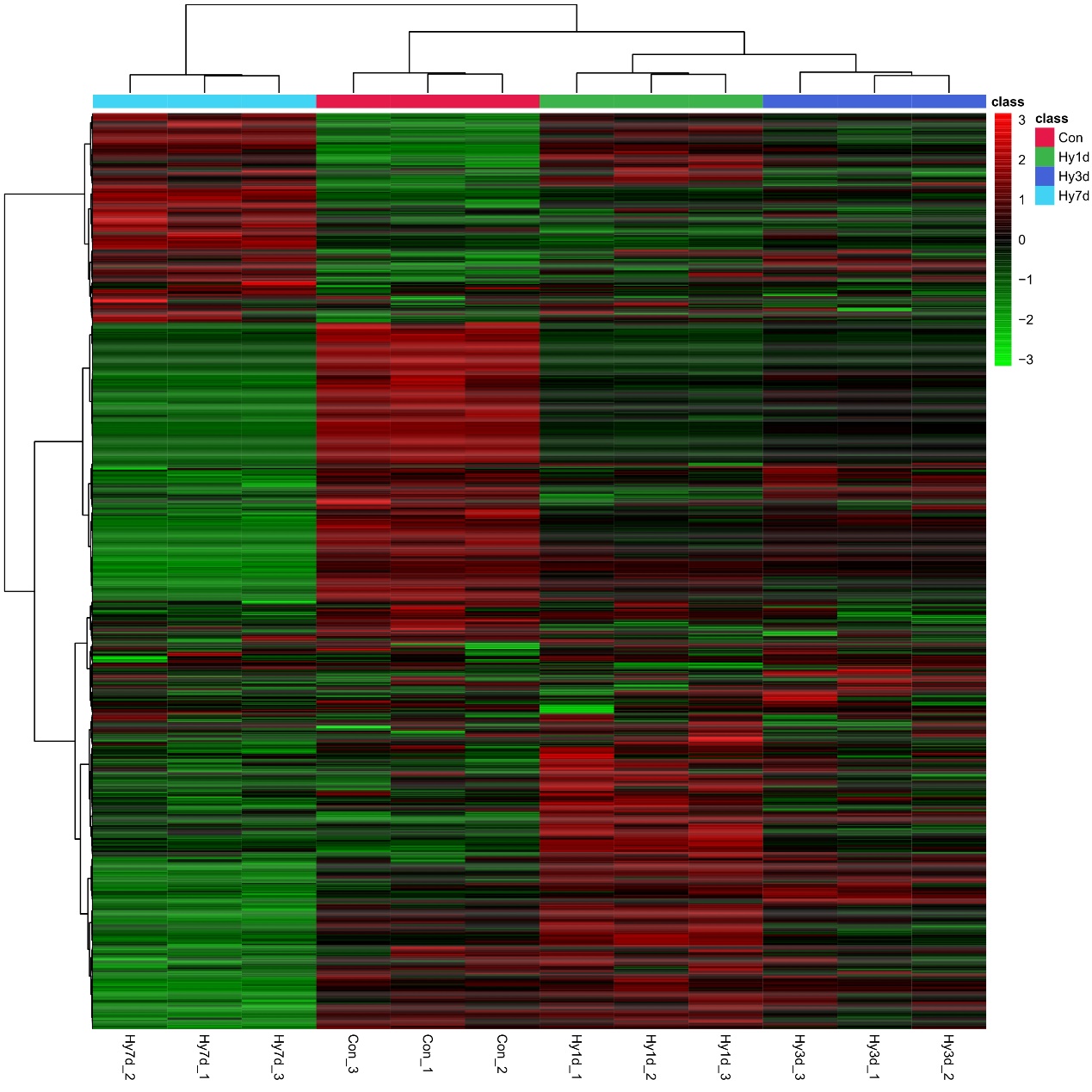


**Figure S2.** Heatmaps of the annotated lipids of mouse hippocampal tissue after 1, 3 and 7 days of hypobaric hypoxia exposure.


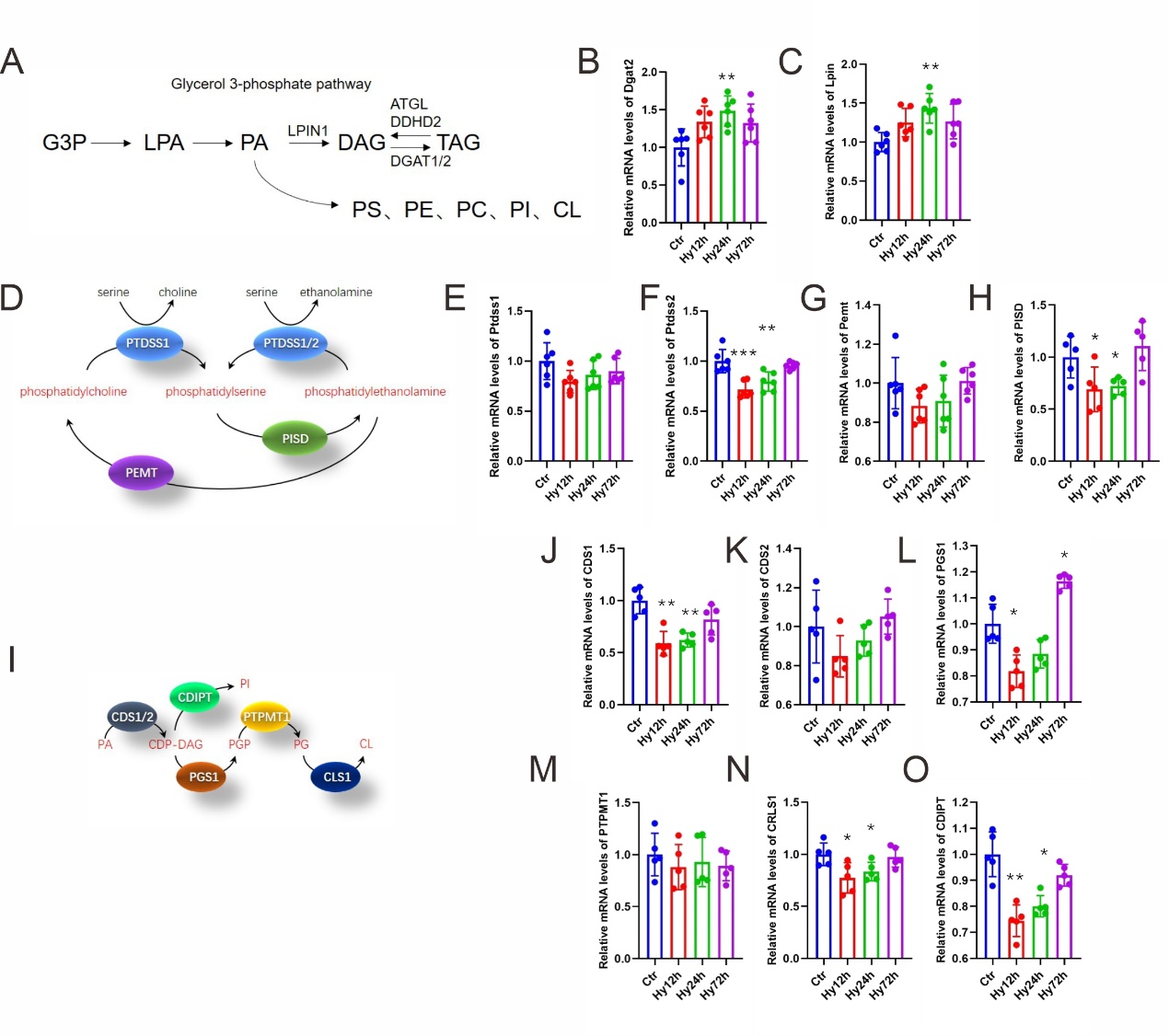


**Figure S3.** Detection of mRNA levels of enzymes related to lipid metabolism.


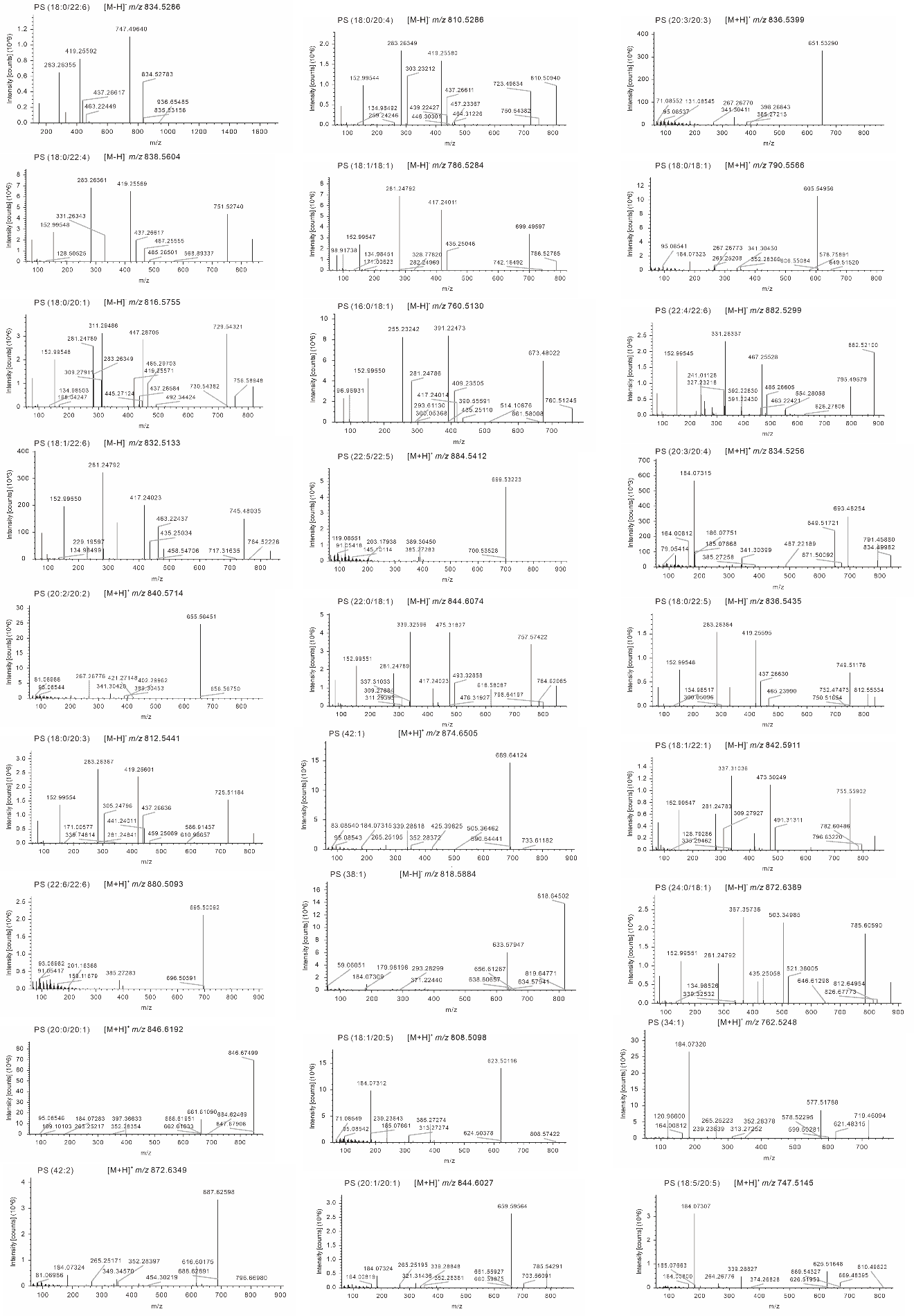


**Figure S4.** MS/MS spectra of PS species acquired in negative/positive ion mode. Related to Table 1-3.


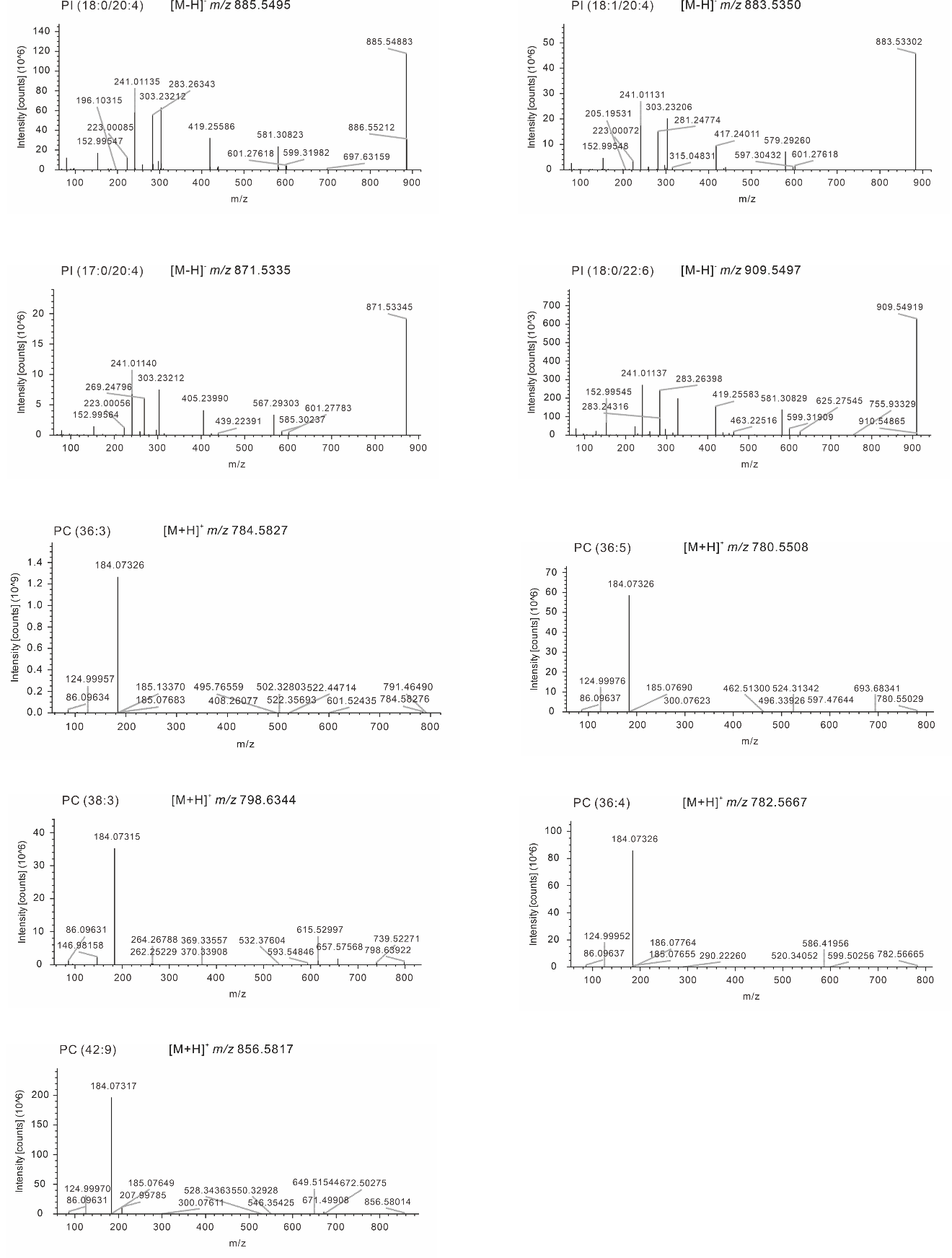


**Figure S5.** MS/MS spectra of PI and PC species acquired in negative/positive ion mode. Related to Table 1-3.


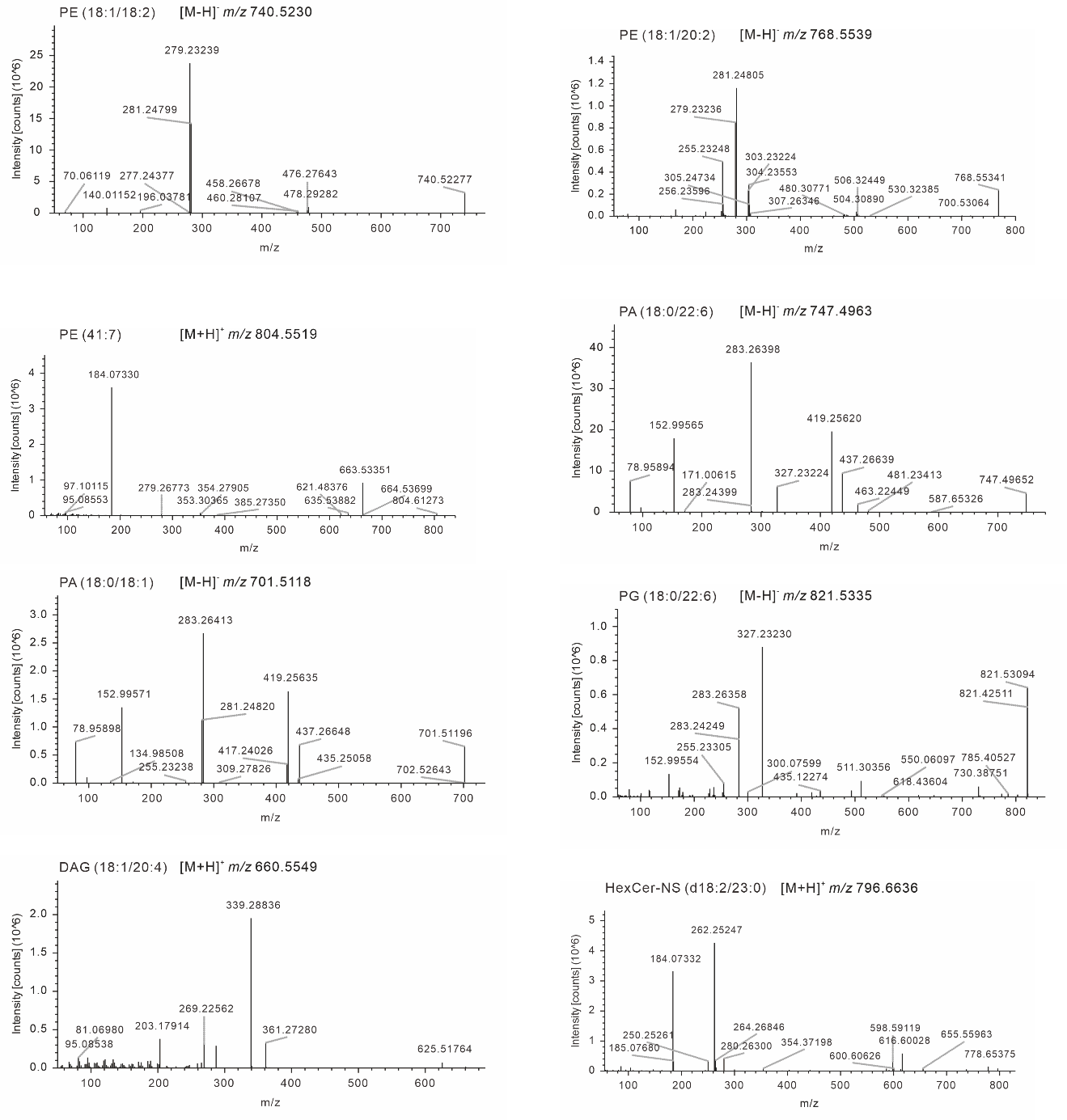


**Figure S6.** MS/MS spectra of PE, PA, PG, DAG and Hex-NS species acquired in negative/positive ion mode. Related to Table 1-3.


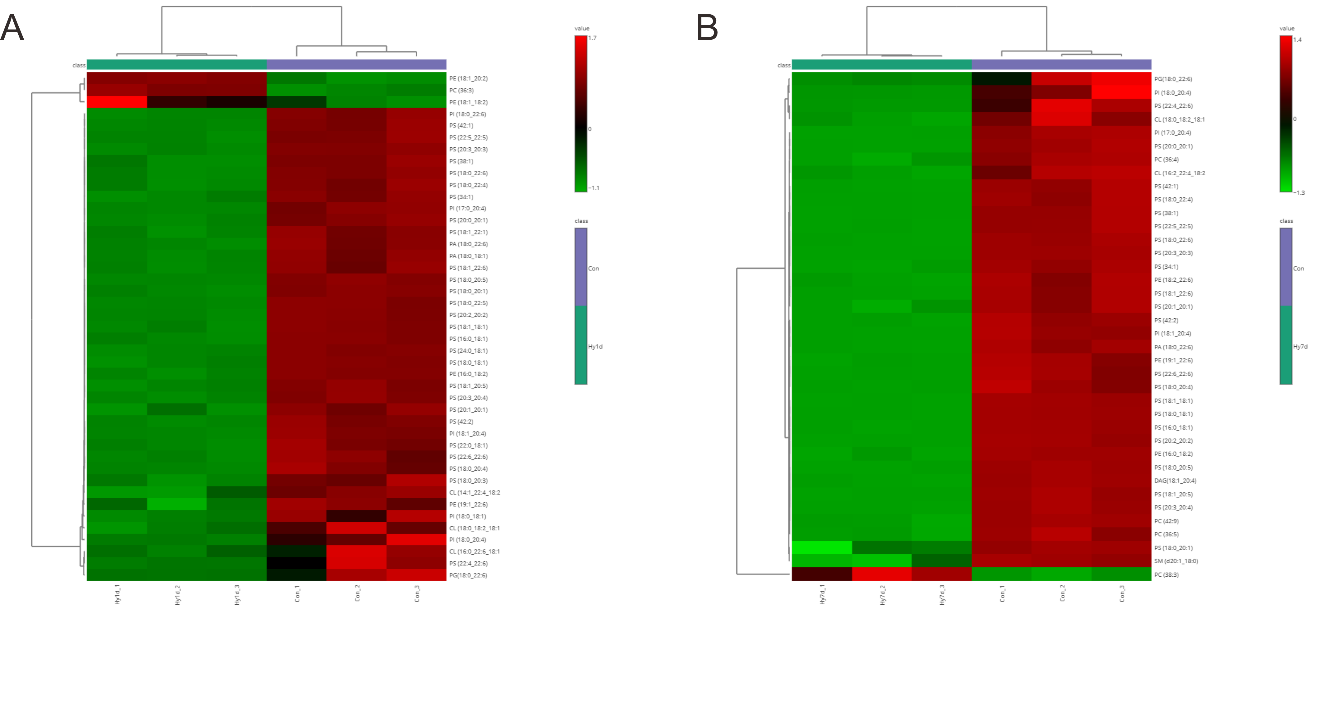


**Figure S7.** Heatmaps of the screened differential lipids of mouse hippocampal tissue after 1 (A) and 7 (B) days of hypobaric hypoxia exposure.


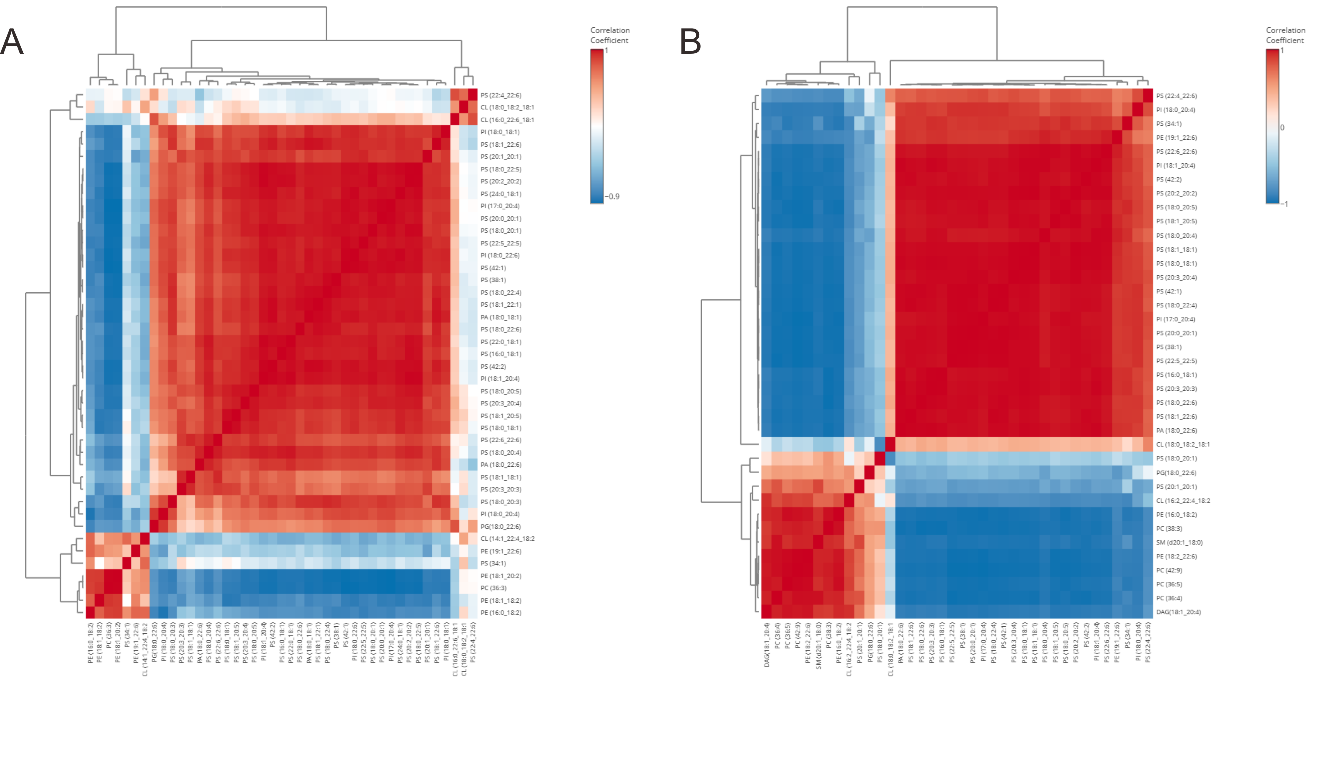


**Figure S8.** Correlation coefficient graphs of the screened differential lipids of mouse hippocampal tissue after 1 (A) and 7 (B) days of hypobaric hypoxia exposure.

**Table S1** Primer sequences used for RT-qPCR.

| Gene Name | Forward Primer (5’-3’) | Reverse Primer(5’-3’) |
| --- | --- | --- |
| *β-Actin* | GAGAGGTTCCGTTGCCCAGAG | CAGACAGCACAGTGTTGGCGT |
| *Dgat2* | CTTCTCTGTCACCTGGCTCA | TGTTCCAGTCAAATGCCAGC |
| *Lpin* | GGAGCTGCGAGAATGGAAAG | GCCTTCCTCCTCCTTTTCCT |
| *Ptdss1* | TTCCTGTGTCTGTACGGCAT | AATGGCTTTCGTGGTTGCTT |
| *Ptdss2* | AGGCTGGTCTTCTTCGTGAA | TGTAGAAGGGCAGTGACAGG |
| *Pemt* | CGGCAATATCGACTTCAGGC | AGTTCTCTGCTCCCATCTCG |
| *Pisd* | TGAGGTGGAGCAGGTAAAGG | GGAGTGGAAGCAGTGGTAGT |
| *Cds1* | TGAGCTTGTACCCCTTCCAG | TGTCCATTATCCCGCCATGT |
| *Cds2* | CTGTGAGCCGTCTGACCTAT | GAATCCACTGGCGAAGAACC |
| *Pgs1* | GGCTGTTATTGGCTCCCTTG | CATCACAACTCGCCTCTTGG |
| *Ptpmt1* | CTGCCGCTGAAGAACATGAC | TGCCTGGTACTTGAGAGCAA |
| *Crls1* | CCGCCAGCTCGTATGAAAAT | GGCCCAGTTTCGAGCAATAA |
| *Cdipt* | CACCCTTCTCTTCCAGCTCA | TCATTTCCAGCACACAGGGT |

**Table S2** Lipid species identified in mice hippocampus related to Table1-3.

| No. | Lipid name | Formula | Acquisition Mode (Positive/Negative) | Molecular Weight | Ret. time (min) | Lipid Subclass | Quant m/z |
| --- | --- | --- | --- | --- | --- | --- | --- |
| 1 | SM (d20:1_18:0) | C43 H87 N2 O6 P | Positive | 758.63 | 12.91 | SM | 759.63 |
| 2 | PS (24:0_18:1) | C48 H92 N O10 P | Negative | 873.65 | 15.00 | PS | 872.64 |
| 3 | PS (22:6_22:6) | C50 H74 N O10 P | Positive | 879.50 | 8.73 | PS | 880.51 |
| 4 | PS (22:5_22:5) | C50 H78 N O10 P | Positive | 883.53 | 9.66 | PS | 884.54 |
| 5 | PS (22:4_22:6) | C50 H78 N O10 P | Negative | 883.54 | 9.67 | PS | 882.53 |
| 6 | PS (22:0_18:1) | C46 H88 N O10 P | Negative | 845.61 | 14.29 | PS | 844.61 |
| 7 | PS (42:2) | C48 H90 N O10 P | Positive | 871.63 | 14.16 | PS | 872.63 |
| 8 | PS (42:1) | C48 H92 N O10 P | Positive | 873.64 | 14.99 | PS | 874.65 |
| 9 | PS (20:3_20:4) | C46 H76 N O10 P | Positive | 833.52 | 9.89 | PS | 834.53 |
| 10 | PS (20:3_20:3) | C46 H78 N O10 P | Positive | 835.53 | 10.93 | PS | 836.54 |
| 11 | PS (20:2_20:2) | C46 H82 N O10 P | Positive | 839.56 | 12.04 | PS | 840.57 |
| 12 | PS (20:1_20:1) | C46 H86 N O10 P | Positive | 843.60 | 13.27 | PS | 844.60 |
| 13 | PS (20:0_20:1) | C46 H88 N O10 P | Positive | 845.61 | 14.26 | PS | 846.62 |
| 14 | PS (38:1) | C44 H84 N O10 P | Positive | 817.58 | 13.34 | PS | 818.59 |
| 15 | PS (18:1_22:6) | C46 H76 N O10 P | Negative | 833.52 | 9.92 | PS | 832.51 |
| 16 | PS (18:1_22:1) | C46 H86 N O10 P | Negative | 843.60 | 13.27 | PS | 842.59 |
| 17 | PS (18:1_20:5) | C44 H74 N O10 P | Positive | 807.50 | 9.82 | PS | 808.51 |
| 18 | PS (18:1_18:1) | C42 H78 N O10 P | Negative | 787.54 | 11.15 | PS | 786.53 |
| 19 | PS (18:0_22:6) | C46 H78 N O10 P | Negative | 835.54 | 10.95 | PS | 834.53 |
| 20 | PS (18:0_22:5) | C46 H80 N O10 P | Negative | 837.55 | 11.73 | PS | 836.54 |
| 21 | PS (18:0_22:4) | C46 H82 N O10 P | Negative | 839.57 | 12.03 | PS | 838.56 |
| 22 | PS (18:0_20:5) | C44 H76 N O10 P | Positive | 746.51 | 10.17 | PS | 747.51 |
| 23 | PS (18:0_20:4) | C44 H78 N O10 P | Negative | 811.54 | 11.26 | PS | 810.53 |
| 24 | PS (18:0_20:3) | C44 H80 N O10 P | Negative | 813.55 | 11.72 | PS | 812.54 |
| 25 | PS (18:0_20:1) | C44 H84 N O10 P | Negative | 817.58 | 13.29 | PS | 816.58 |
| 26 | PS (18:0_18:1) | C42 H80 N O10 P | Positive | 789.55 | 12.27 | PS | 790.56 |
| 27 | PS (34:1) | C40 H76 N O10 P | Positive | 761.52 | 11.06 | PS | 762.52 |
| 28 | PS (16:0_18:1) | C40 H76 N O10 P | Negative | 761.52 | 11.05 | PS | 760.51 |
| 29 | PI (18:1_20:4) | C47 H81 O13 P | Negative | 884.54 | 10.07 | PI | 883.54 |
| 30 | PI (18:0_22:6) | C49 H83 O13 P | Negative | 910.56 | 10.86 | PI | 909.55 |
| 31 | PI (18:0_20:4) | C47 H83 O13 P | Negative | 886.56 | 11.16 | PI | 885.55 |
| 32 | PI (18:0_18:1) | C45 H85 O13 P | Negative | 864.57 | 12.15 | PI | 863.57 |
| 33 | PI (17:0_20:4) | C46 H81 O13 P | Negative | 872.54 | 10.57 | PI | 871.53 |
| 34 | PG(18:0_22:6) | C46 H79 O10 P | Negative | 822.54 | 11.02 | PG | 821.53 |
| 35 | PE (41:7) | C46 H78 N O8 P | Positive | 803.54 | 11.32 | PE | 804.55 |
| 36 | PE (18:2_22:6) | C45 H74 N O8 P | Negative | 787.52 | 9.90 | PE | 786.51 |
| 37 | PE (18:1_20:2) | C43 H80 N O8 P | Negative | 769.56 | 10.88 | PE | 768.55 |
| 38 | PE (18:1_18:2) | C41 H76 N O8 P | Negative | 741.53 | 11.11 | PE | 740.52 |
| 39 | PE (16:0_18:2) | C39 H74 N O8 P | Negative | 715.51 | 11.12 | PE | 714.51 |
| 40 | PC (42:9) | C50 H82 N O8 P | Positive | 855.57 | 9.89 | PC | 856.58 |
| 41 | PC (36:4) | C44 H80 N O8 P | Positive | 781.56 | 10.04 | PC | 782.57 |
| 42 | PC (36:3) | C44 H82 N O8 P | Positive | 783.58 | 10.88 | PC | 784.58 |
| 43 | PC (36:5) | C44 H78 N O8 P | Positive | 779.54 | 9.97 | PC | 780.55 |
| 44 | PC (38:3) | C46 H88 N O7 P | Positive | 797.63 | 13.41 | PC | 798.63 |
| 45 | PA (18:0_22:6) | C43 H73 O8 P | Negative | 748.50 | 10.96 | PA | 747.50 |
| 46 | PA (18:0_18:1) | C39 H75 O8 P | Negative | 702.52 | 12.26 | PA | 701.51 |
| 47 | HexCer_NS (d18:2_23:0) | C47 H89 N O8 | Positive | 795.66 | 13.92 | GlcCer | 796.66 |
| 48 | DAG(18:1_20:4) | C41 H70 O5 | Positive | 659.55 | 12.62 | DAG | 660.55 |
| 49 | CL (18:0_18:2_18:1_20:3) | C83 H150 O17 P2 | Positive | 1481.03 | 16.50 | CL | 1482.04 |
| 50 | CL (16:2_22:4_18:2_20:3) | C85 H144 O17 P2 | Positive | 1516.01 | 15.88 | CL | 1517.02 |
| 51 | CL (16:0_22:6_18:1_18:2) | C83 H144 O17 P2 | Positive | 1492.01 | 15.98 | CL | 1493.02 |
| 52 | CL (14:1_22:4_18:2_20:3) | C83 H142 O17 P2 | Positive | 1490.00 | 15.82 | CL | 1491.01 |
